# Comparison of the associations between office and home BP with placenta-mediated pregnancy complications: the BOSHI study

**DOI:** 10.1038/s41440-025-02439-x

**Published:** 2025-11-20

**Authors:** Noriyuki Iwama, Seiya Izumi, Hiroki Nobayashi, Takahisa Murakami, Michihiro Satoh, Hasumi Tomita, Hirotaka Hamada, Mami Ishikuro, Taku Obara, Masatoshi Saito, Takayoshi Ohkubo, Shinichi Kuriyama, Kazuhiko Hoshi, Yutaka Imai, Hirohito Metoki, The BOSHI Study Group

**Affiliations:** 1 Center for Perinatal Medicine, Tohoku University Hospital, Sendai, Miyagi Japan; 2 Department of Preventive Medicine and Epidemiology, Tohoku Medical Megabank Organization, Tohoku University, Sendai, Miyagi Japan; 3 Department of Obstetrics and Gynecology, Tohoku University Graduate School of Medicine, Sendai, Miyagi Japan; 4 Division of Public Health, Hygiene and Epidemiology, Tohoku Medical Pharmaceutical University, Sendai, Miyagi Japan; 5 Division of Nephrology and Hypertension, Department of Internal Medicine, The Jikei University School of Medicine, Tokyo, Japan; 6 Department of Pharmacy, Tohoku Medical and Pharmaceutical University Hospital, Sendai, Miyagi Japan; 7 Division of Molecular Epidemiology, Tohoku University Graduate School of Medicine, Sendai, Miyagi Japan; 8 Department of Maternal and Fetal Therapeutics, Tohoku University Graduate School of Medicine, Sendai, Miyagi Japan; 9 Department of Hygiene and Public Health, Teikyo University School of Medicine, Tokyo, Japan; 10 Tohoku Institute for Management of Blood Pressure, Sendai, Miyagi Japan; 11 Environment and Genome Research Center, Tohoku University Graduate School of Medicine, Sendai, Miyagi Japan; 12 International Research Institute of Disaster Science, Tohoku University, Sendai, Miyagi Japan; 13 Suzuki Memorial Hospital, Iwanuma, Miyagi Japan

**Keywords:** Home blood pressure, hypertensive disorders of pregnancy, office blood pressure, morning hypertension, placenta-mediated pregnancy complications

## Abstract

This prospective cohort study compared the associations between office blood pressure (OBP) and home blood pressure (HBP) measured before 20 weeks of gestation with the subsequent development of placenta-mediated pregnancy complications (PMPCs). A total of 975 pregnant women were included in the study. OBP and HBP were measured between 10 weeks 0 days and 19 weeks 6 days of gestation, using HBP values from the same gestational weeks as OBP. When both OBP and HBP were included simultaneously in a binary logistic regression model, per 1 standard deviation increase, the adjusted odds ratios (aORs) for office and home systolic blood pressure (SBP) were 1.16 (95% confidence interval [CI]: 0.95–1.42) and 1.68 (95% CI: 1.36–2.09), respectively. For diastolic blood pressure (DBP), the aORs were 1.36 (95% CI: 1.10–1.69) for office and 1.70 (95% CI: 1.37–2.12) for home measurements. The likelihood ratio test showed that adding home SBP to a model with office SBP improved model fit (P value < 0.0001), whereas adding office SBP to a model with home SBP did not (P value = 0.2). For DBP, adding either home or office values improved model fit (P value < 0.0001 and P value = 0.005, respectively). Home SBP was more strongly associated with PMPCs than office SBP. Although home DBP was not statistically stronger than office DBP, its effect estimate was higher. These findings support the added value of HBP monitoring during pregnancy for predicting PMPCs.

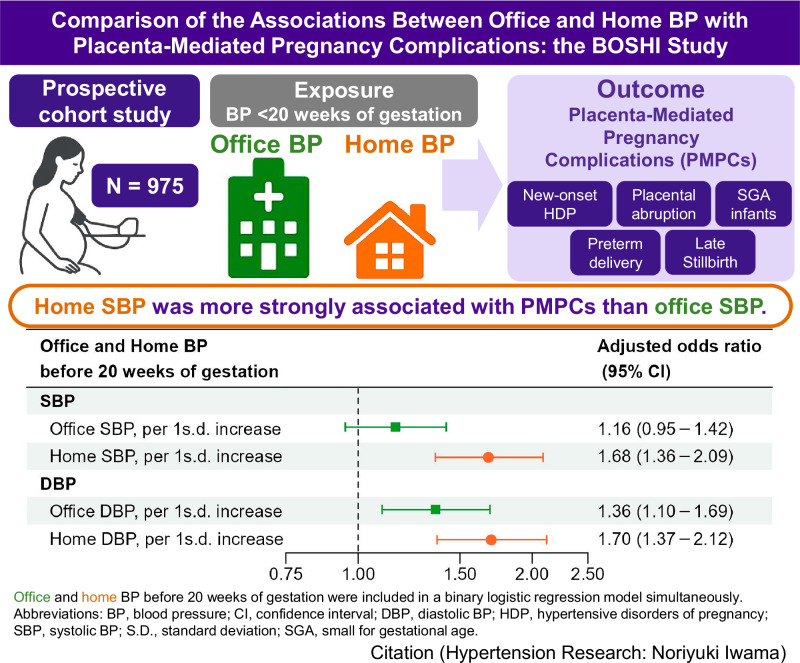

## Introduction

Blood pressure (BP) monitoring is fundamental to cardiovascular risk management. In nonpregnant adults, out-of-office measurements—particularly home blood pressure (HBP)—have been shown to provide superior prognostic value compared with office blood pressure (OBP), largely because they minimize white-coat effects and capture day-to-day variability [1, 2]. These advantages have led to the widespread adoption of HBP monitoring in clinical guidelines [3–5].

In pregnancy, BP assessment is essential for identifying women at risk of placenta-mediated pregnancy complications (PMPCs), which include hypertensive disorders of pregnancy (HDP)—such as gestational hypertension and preeclampsia—as well as placental abruption, preterm delivery, small for gestational age (SGA) infants, and stillbirth at ≥28 weeks of gestation [6, 7]. These complications are believed to result from abnormal placentation and impaired maternal vascular adaptation to pregnancy [8, 9]. PMPCs are a leading cause of maternal and perinatal morbidity and mortality worldwide [10–12] and are associated not only with adverse perinatal outcomes but also with long-term cardiovascular and metabolic diseases in mothers [13, 14].

While maternal HBP levels during pregnancy have been increasingly evaluated in antenatal care, their prognostic value relative to OBP in predicting PMPCs remains insufficiently established [15–17]. To date, only one study has directly compared maternal OBP and HBP levels before 20 weeks of gestation in relation to perinatal outcomes. In that study, higher HBP levels before 20 weeks of gestation were more strongly associated with lower infant birth weight than OBP—infant birth weight being one component of perinatal outcomes—suggesting that HBP levels before 20 weeks of gestation may more accurately reflect maternal hemodynamic status during pregnancy [18]. However, no study to date has directly compared maternal OBP and HBP levels in relation to the risk of PMPCs, and it remains unclear whether HBP measurement during pregnancy can improve the prediction of PMPCs in addition to OBP. Addressing this knowledge gap is important, as early identification of pregnant women at high risk of PMPCs is essential for implementing timely interventions, such as low-dose aspirin, folic acid, and multivitamin supplementation, or closer surveillance, which may mitigate adverse outcomes [19–25].

This study aimed to compare the associations of maternal OBP and HBP measured before 20 weeks of gestation with the development of PMPCs, using data from a prospective cohort study. We hypothesized that maternal HBP levels during pregnancy would demonstrate stronger associations with PMPCs than OBP, supporting the integration of HBP monitoring into routine prenatal checkups.

## Methods

### Study design and participants

This study used data from the Babies and their parents’ longitudinal Observation in Suzuki memorial Hospital on Intrauterine period (BOSHI) study, a prospective cohort study conducted at Suzuki Memorial Hospital—an obstetrics and gynecology facility located in Sendai City, Miyagi Prefecture, Japan. The study protocol was approved by the Institutional Review Board of the Tohoku University School of Medicine (No. 2019-7) and the Ethics Committee of Suzuki Memorial Hospital. Written informed consent was obtained from all participants. Further details of the study have been published elsewhere [26–29].

### Maternal OBP and HBP measurement during pregnancy

Maternal OBP was measured using the HEM-705IT device (Omron Healthcare, Kyoto, Japan), a digital automated sphygmomanometer that employs the cuff-oscillometric method. This device provides digital readings of systolic blood pressure (SBP) and diastolic blood pressure (DBP), and uses the same algorithm that are validated for use in pregnant women [30]. In Japan, routine antenatal checkups are conducted every four weeks until 23 weeks of gestation, every two weeks from 24 to 35 weeks of gestation, and weekly from 36 weeks of gestation onward. During each visit, participants were instructed to rest in a seated position for 1–2 min before undergoing two consecutive blood pressure measurements. For this study, at least one OBP reading per visit was required; if two readings were available, their average was used for analysis.

Maternal home blood pressure (HBP) was measured in accordance with the Japanese Society of Hypertension guidelines for home blood pressure monitoring [31]. Participants used either the HEM-747IC or HEM-7080IC (Omron Healthcare, Kyoto, Japan), both of which use the same algorithm as the HEM-705IT and are validated for use in pregnant women [30]. HBP was measured at the upper arm every morning within one hour of waking, after urination, before breakfast, while seated, and after resting for at least one minute. Detailed OBP and HBP measurement protocols in the BOSHI study have been described in previous publications [26, 28].

Because the number of OBP and HBP measurements obtained before 10 weeks of gestation was insufficient for analysis, these early readings were excluded. Prior studies have reported that elevated OBP—even before 20 weeks of gestation, when new-onset HDP are typically diagnosed—is associated with increased risks of both SGA infants and HDP. Previous research has shown that elevated OBP and HBP levels between 10 weeks 0 days and 19 weeks 6 days of gestation are associated with lower infant birth weights [18]. Therefore, this study focused on OBP and HBP measurements obtained during this gestational period. To enable direct comparison between OBP and HBP levels, we used HBP readings taken during the same gestational weeks as the corresponding OBP measurements. For each participant, a one-week average of HBP values was calculated. When multiple OBP measurements and corresponding one-week HBP average values were available within 10 weeks 0 days and 19 weeks 6 days of gestation, the earliest measurement was used. Mean arterial pressure (MAP) for both office and home measurements was calculated as follows: MAP = (SBP − DBP) / 3 + DBP.

### Definition of PMPCs

The primary outcome of this study, PMPCs, was defined as the occurrence of at least one of the following perinatal outcomes: new-onset HDP (gestational hypertension or preeclampsia), placental abruption, preterm delivery, SGA infants, and stillbirth at ≥28 weeks of gestation [6, 7]. Because the study focused on new-onset hypertension during pregnancy, women who had chronic hypertension or superimposed preeclampsia were excluded. New-onset HDP were defined according to the guidelines of the Japan Society for the Study of Hypertension in Pregnancy, based on OBP measurements [32]. Specifically, new-onset HDP was defined as the occurrence of hypertension (OBP ≥ 140/90 mmHg) occurring between 20 weeks of gestation and 12 weeks postpartum. New-onset HDP was further classified into gestational hypertension and preeclampsia. Gestational hypertension was defined as sustained hypertension without proteinuria, whereas preeclampsia was defined as new-onset hypertension accompanied by proteinuria (≥300 mg/day) within the same period.

Information on placental abruption and stillbirth at ≥28 weeks of gestation, gestational age at delivery, infant sex, and infant birth weight was obtained from obstetric medical records. Preterm delivery was defined as delivery between 22 weeks 0 days and 36 weeks 6 days of gestation. This study did not collect data allowing for differentiation between spontaneous and iatrogenic preterm delivery. Infant birth weight percentiles in Japan are customized based on parity, gestational age at delivery, infant sex, and infant birth weight [33]. This study defined SGA infants as those with birth weight below the 10th percentile for gestational age.

### Other variables used in this study

Details of other variables used in this study were provided in the Supplementary Information.

### Statistical analyses

To examine the associations of OBP and HBP with PMPCs, a binary logistic regression model was employed to estimate odds ratios (ORs). Initially, OBP and HBP were included in the model separately. Each was categorized into quartiles and included as a categorical variable. To evaluate linear trends, quartiles were also treated as continuous variables, and the P value for trend was calculated. Subsequently, 1 standard deviation (s.d.) of OBP or HBP was included in the model as a continuous variable separately. Finally, both OBP and HBP (each per 1-s.d. increase) were included in the model as continuous variables simultaneously to compare the associations between OBP and HBP with PMPCs. Model fit was compared using the likelihood ratio test between the models including OBP or HBP separately and models including both OBP and HBP simultaneously.

Two models were constructed in a binary logistic regression model. Model 1 was unadjusted, whereas Model 2 was adjusted for maternal age, pre-pregnancy body mass index, parity, assisted reproductive technology, family history of hypertension, smoking status, alcohol intake, HbA1c level before 20 weeks of gestation, and infant sex, with reference to previous studies [34–41]. In addition, the season of estimated date of confinement and gestational age when BP was measured were included to account for seasonal and physiological BP changes during pregnancy [17, 26, 28, 42]. A directed acyclic graph illustrating the associations of OBP and HBP with PMPCs is presented in Supplementary Fig. 1. Previous study indicated that gestational weight gain (GWG) was associated with SGA infants, one of the components of PMPCs. GWG was not included as a covariate because it was considered an intervening variable in this study [43].

Multicollinearity was assessed using variance inflation factors (VIF), with values ≥4.0 indicating potential concern. VIFs were calculated using a general linear model with PMPCs as the dependent variable and the same covariates as those in the logistic regression models. Because several covariates had missing values with a non-monotone pattern, multivariate imputation by chained equations (MICE) was performed. Ten imputed datasets were generated, and the analyses were repeated in each. The results were combined using Rubin’s rules [44]. The D3 statistic, a pooled likelihood ratio test statistic across multiple imputations, was also calculated in Model 2 [44].

As an additional analysis, we compared the associations of SBP and DBP with PMPCs, separately for OBP and HBP measurements. Both SBP and DBP (per 1 s.d. increase) were included simultaneously as continuous variables in a binary logistic regression model. Model fit was compared using the likelihood ratio between models, including SBP or DBP separately, and the model including both SBP and DBP simultaneously.

For sensitivity analysis, PMPCs were redefined by replacing new-onset HDP with preeclampsia, and the analysis was conducted using this alternative definition.

A two-sided P value of <0.05 was considered statistically significant. Statistical analyses were performed using the SAS software version 9.4 (SAS Institute Inc., Cary, North Carolina, USA) and R version 4.4.2 (R Foundation, Vienna, Austria) [45].

## Results

### Maternal and neonatal characteristics of the study participants

**Fig. 1:**
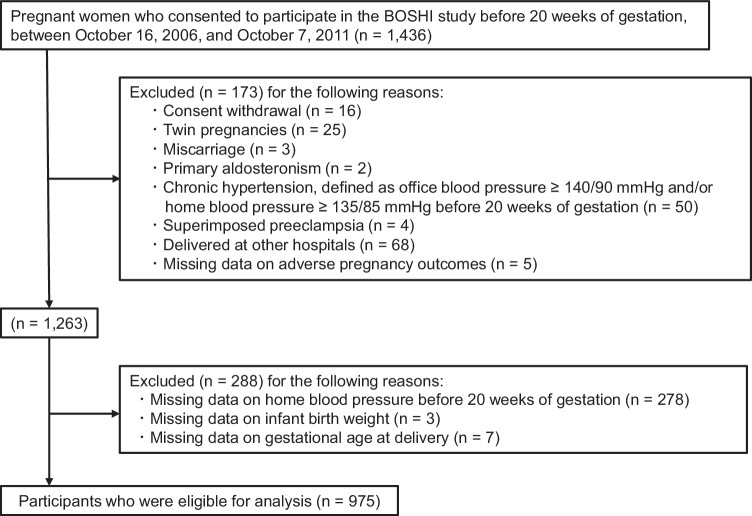
Flowchart illustrating the enrollment and the inclusion process of study participants

A flowchart illustrating participant enrollment and inclusion is presented in Fig. 1. A total of 1,436 pregnant women provided consent to participate in the BOSHI study before 20 weeks of gestation between October 16, 2006, and October 7, 2011. Participants were excluded for the following reasons: consent withdrawal (n = 16), twin pregnancies (n = 25), miscarriage (n = 3), primary aldosteronism (n = 2), chronic hypertension—defined as OBP ≥ 140/90 mmHg and/or HBP ≥ 135/85 mmHg before 20 weeks of gestation (n = 50). Additional exclusions were made for superimposed preeclampsia (n = 4), delivery at other hospitals (n = 68), and missing data on adverse pregnancy outcomes (n = 5). Participants with missing data on HBP before 20 weeks of gestation (n = 278), infant birth weight (n = 3), or gestational age at delivery (n = 7) were also excluded. Ultimately, 975 participants were eligible for analysis.

Table 1 summarizes the maternal and neonatal characteristics of the study participants. The mean (s.d.) gestational ages at OBP and HBP measurement were 13.9 (2.3) weeks and 14.0 (2.2) weeks, respectively. The number and proportion of participants according to the number of OBP and HBP measurements are presented in Supplementary Table 1. The number and percentage of PMPC cases were 170 (17.4%). New-onset HDP represented the most common complication among the composite outcomes of PMPC in participants who developed the condition. Supplementary Tables 2–7 present maternal and neonatal characteristics according to quartiles of each OBP and HBP measure.

**Table 1:** Maternal and neonatal characteristics of study participants

Variables	All participants (N = 975)
**Maternal age, years (N** = **973)**	31.3 (4.7)
<35 years, N (%)	716 (73.4)
≥35 years, N (%)	257 (26.4)
Missing, N (%)	2 (0.2)
**Height, cm**	158.5 (5.2)
**Pre-pregnancy body weight, kg**	54.0 (8.5)
**Pre-pregnancy BMI, kg/m²**	21.5 (3.1)
Underweight (<18.5 kg/m²), N (%)	124 (12.7)
Normal weight (18.5–24.9 kg/m²), N (%)	735 (75.4)
Overweight/Obese (≥25.0 kg/m²), N (%)	116 (11.9)
**Parity, N (%)**	
Primipara	587 (60.2)
Multipara without HDP in a previous pregnancy	356 (36.5)
Multipara with HDP in a previous pregnancy	20 (2.1)
Missing	12 (1.2)
**ART, N (%)**	27 (2.8)
**Family history of hypertension, N (%)**	50 (5.1)
**Smoking status, N (%)**	
No smoking before conception	824 (84.5)
Until conception was recognized	120 (12.3)
Smoking during pregnancy	31 (3.2)
**Alcohol drinking, N (%)**	
No alcohol intake before conception	518 (53.1)
Until conception was recognized	349 (35.8)
Alcohol intake during pregnancy	99 (10.2)
Missing	9 (0.9)
**HbA1c before 20 weeks of gestation, % (N** = **957)**	5.0 (0.3)
**Season of estimated date of confinement, N (%)**	
Spring	242 (24.8)
Summer	246 (25.2)
Autumn	252 (25.8)
Winter	235 (24.1)
**Hyperthyroidism, N (%)**	7 (0.7)
**Hypothyroidism, N (%)**	2 (0.2)
**Chronic kidney disease, N (%)**	0 (0.0)
**Gestational age when office BP was measured, weeks**	13.9 (2.3)
**Gestational age when home BP was measured, weeks**	14.0 (2.2)
**Number of office BP measurements, median (range)**	2 (1–2)
**Number of home BP measurements, median (range)**	3 (1–7)
**Office SBP before 20 weeks of gestation, mmHg**	108 (10)
**Office DBP before 20 weeks of gestation, mmHg**	66 (8)
**Office MAP before 20 weeks of gestation, mmHg**	80 (8)
**Home SBP before 20 weeks of gestation, mmHg**	104 (9)
**Home DBP before 20 weeks of gestation, mmHg**	63 (7)
**Home MAP before 20 weeks of gestation, mmHg**	77 (7)
**PMPCs, N (%)**	170 (17.4)
New-onset HDP, N (%)	92 (9.4)
Preeclampsia, N (%)	17 (1.7)
Gestational hypertension, N (%)	75 (7.7)
Placental abruption, N (%)	3 (0.3)
Preterm delivery (<37 weeks of gestation), N (%)	29 (3.0)
SGA infants (Birth weight <10th percentile), N (%)	65 (6.7)
Stillbirth at ≥28 weeks of gestation, N (%)	2 (0.2)
**Redefined PMPCs by replacing new-onset HDP with preeclampsia, N (%)**	103 (10.6)
**Infant sex, N (%)**	
Male	507 (52.0)
Female	468 (48.0)
**Delivery week, weeks**	39.6 (1.7)
**Infant birth weight, g**	3,053 (420)
Low birth weight (<2,500 g), N (%)	70 (7.2)

Unless otherwise specified, continuous and categorical variables are expressed as mean (s.d.) and numbers (percentages), respectively

PMPCs include at least one of the following perinatal outcomes: new-onset HDP, placental abruption, spontaneous preterm delivery, SGA infants, and stillbirth at ≥28 weeks of gestation

*ART* assisted reproductive technology, *BMI* body mass index, *BP* blood pressure, *DBP* diastolic blood pressure, *HDP* hypertensive disorders of pregnancy, *MAP* mean arterial pressure, *PMPCs* placenta-mediated pregnancy complications, *SBP* systolic blood pressure, *s.d.* standard deviation, *SGA* small for gestational age

### The association between OBP and PMPCs (Separate analysis)

**Fig. 2:**
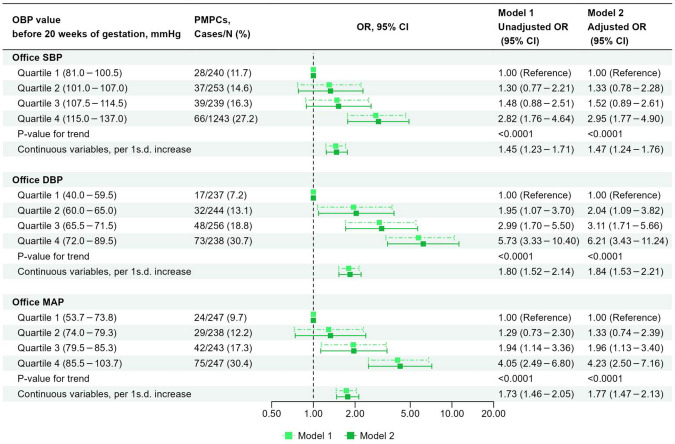
The association between OBP and PMPCs (separate analysis). Model 1: Unadjusted model. Model 2: Adjusted for maternal age, pre-pregnancy BMI, parity, ART, family history of hypertension, smoking status, alcohol intake, HbA1c level before 20 weeks of gestation, season of EDC, infant sex, and gestational age when OBP was measured. ART assisted reproductive technology, BP blood pressure, BMI body mass index, CI confidence interval, DBP diastolic BP, EDC estimated date of confinement, HDP hypertensive disorders of pregnancy, MAP mean arterial pressure, OBP office BP, OR odds ratio, PMPCs placenta-mediated pregnancy complications, SBP systolic BP, s.d. standard deviation, SGA small for gestational age. 1 s.d. = 10 mmHg for office SBP, 8 mmHg for office DBP, and 8 mmHg for office MAP

Figure 2 shows the association between OBP and PMPCs. A higher office SBP, DBP, and MAP were associated with increased odds of PMPCs in Model 2 (P value for trend was <0.0001 for all). The adjusted ORs for the occurrence of PMPCs associated with a 1 s.d. increase in office SBP, DBP, and MAP were 1.47 (95% confidence interval [CI]: 1.24–1.76), 1.84 (95% CI: 1.53–2.21), and 1.77 (95% CI: 1.47–2.13), respectively.

### The association between HBP and PMPCs (Separate analysis)

**Fig. 3:**
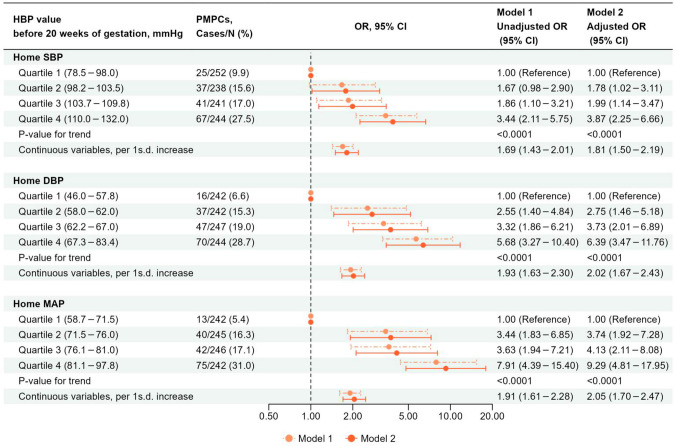
The association between HBP and PMPCs (separate analysis). Model 1: Unadjusted model. Model 2: Adjusted for maternal age, pre-pregnancy BMI, parity, ART, family history of hypertension, smoking status, alcohol intake, HbA1c level before 20 weeks of gestation, season of EDC, infant sex, and gestational age when HBP was measured. ART assisted reproductive technology, BP blood pressure, BMI body mass index, CI confidence interval, DBP diastolic BP, EDC estimated date of confinement, HBP home BP, MAP mean arterial pressure, OR odds ratio, PMPCs placenta-mediated pregnancy complications, SBP systolic BP, s.d. standard deviation, SGA small for gestational age. 1 s.d. = 9 mmHg for home SBP, 7 mmHg for home DBP, and 7 mmHg for home MAP

Figure 3 shows the association between HBP and PMPCs. A higher home SBP, DBP, and MAP were associated with increased odds of PMPCs in Model 2 (P value for trend was <0.0001 for all). The adjusted ORs for the occurrence of PMPCs associated with a 1 s.d. increase in home SBP, DBP, and MAP were 1.81 (95% CI: 1.50–2.19), 2.02 (95% CI: 1.67–2.43), and 2.05 (95% CI: 1.70–2.47), respectively.

### Comparison of the associations between OBP and HBP with PMPCs (Simultaneous analysis)

**Fig. 4:**
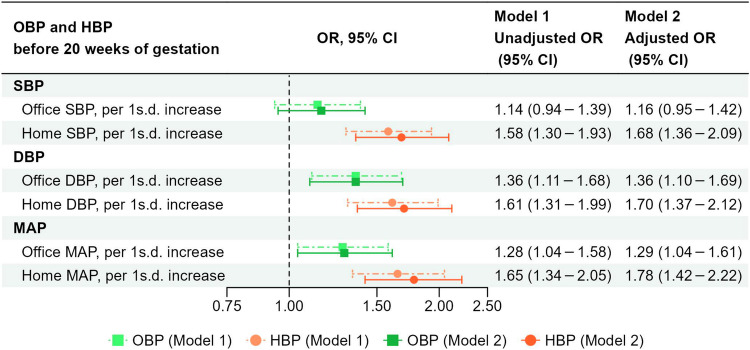
Comparison of the associations between OBP and HBP with PMPCs (simultaneous analysis). In the simultaneous analysis, both OBP and HBP were included in a binary logistic regression model, as continuous variables simultaneously per 1 s.d. Model 1: Unadjusted model. Model 2: Adjusted for maternal age, pre-pregnancy BMI, parity, ART, family history of hypertension, smoking status, alcohol intake, HbA1c level before 20 weeks of gestation, season of EDC, infant sex, and gestational age when OBP was measured. ART assisted reproductive technology, BP blood pressure, BMI body mass index, CI confidence interval, DBP diastolic BP, EDC estimated date of confinement, HBP home BP, MAP mean arterial pressure, OBP office BP, OR odds ratio, PMPCs placenta-mediated pregnancy complications, SBP systolic BP, s.d. standard deviation. 1 s.d. = 10 mmHg for office SBP, 8 mmHg for office DBP, and 8 mmHg for office MAP. 1 s.d. = 9 mmHg for home SBP, 7 mmHg for home DBP, and 7 mmHg for home MAP

Figure 4 indicates the comparison of the associations between OBP and HBP with PMPCs. When both office and home SBP were included simultaneously in the model as continuous variables (per 1 s.d. increase), the association with home SBP remained statistically significant, whereas the association with office SBP was attenuated. In Model 2, the adjusted ORs for the occurrence of PMPCs associated with a 1 s.d. increase in office and home SBP were 1.16 (95% CI: 0.95–1.42) and 1.68 (95% CI: 1.36–2.09), respectively. When both office and home DBP were included simultaneously as continuous variables (per 1 s.d. increase), both remained significantly associated with PMPCs, with the point estimate for home DBP being higher than that for office DBP. The adjusted ORs for PMPCs per 1 s.d. increase in office and home DBP were 1.36 (95% CI: 1.10–1.69) and 1.70 (95% CI: 1.37–2.12), respectively. Similarly, when both office and home MAP were included simultaneously in the model as continuous variables (per 1 s.d. increase), both remained significantly associated with PMPCs, and the point estimate for home MAP was higher than that for office MAP. The adjusted ORs for PMPCs per 1 s.d. increase in office and home MAP were 1.29 (95% CI: 1.04–1.61) and 1.78 (95% CI: 1.42–2.22), respectively.

Table 2 also indicates the likelihood ratio test results. For SBP, adding HBP to a model containing OBP significantly improved model fit (P value was <0.0001), whereas adding OBP to a model containing HBP did not significantly improve model fit (P value = 0.2). For DBP, adding HBP to a model containing OBP significantly improved model fit (P value was <0.0001). Likewise, adding OBP to a model containing HBP also significantly improved model fit (P value = 0.005). For MAP, including HBP in a model with OBP significantly enhanced model fit (P value was <0.0001), and the addition of OBP to a model with HBP also resulted in a statistically significant improvement (P value = 0.02).

**Table 2:** Comparison of the associations between OBP and HBP with PMPCs (Likelihood ratio test)

OBP and/or HBP before 20 weeks of gestation	Model 1 Unadjusted model		Model 2 Adjusted model	
	Likelihood ratio test statistic	P value	D3-statistic	P value
**SBP, per 1 s.d. increase**				
Office SBP + home SBP vs. Office SBP	20.81	<0.0001	24.25	<0.0001
Office SBP + home SBP vs. Home SBP	1.67	0.2	1.96	0.2
**DBP, per 1 s.d. increase**				
Office DBP + home DBP vs. Office DBP	20.67	<0.0001	23.04	<0.0001
Office DBP + home DBP vs. Home DBP	8.52	0.004	8.02	0.005
**MAP, per 1 s.d. increase**				
Office MAP + home MAP vs. Office MAP	22.58	0.0002	26.50	<0.0001
Office MAP + home MAP vs. Home MAP	5.28	0.01	5.18	0.02

The D3-statistic is a pooled likelihood ratio test statistic across multiple imputations

Model 1: Unadjusted model

Model 2: Adjusted for maternal age, pre-pregnancy BMI, parity, ART, family history of hypertension, smoking status, alcohol intake, HbA1c level before 20 weeks of gestation, season of EDC, infant sex, and gestational age when BP was measured

1 s.d. = 10 mmHg for office SBP, 8 mmHg for office DBP, and 8 mmHg for office MAP

1 s.d. = 9 mmHg for home SBP, 7 mmHg for home DBP, and 7 mmHg for home MAP

*ART* assisted reproductive technology, *BP* blood pressure, *BMI* body mass index, *DBP* diastolic BP, *EDC* estimated date of confinement, *HBP* home BP, *MAP* mean arterial pressure, *OBP* office BP, *PMPCs* placenta-mediated pregnancy complications, *SBP* systolic BP, *s.d.* standard deviation

### Comparison of the associations between SBP and DBP with PMPCs (Simultaneous analysis)

Supplementary Figure 2 shows comparison of the associations between SBP and DBP with PMPCs. When office and home SBP and DBP were included simultaneously in the model, the association for SBP was no longer statistically significant, while DBP remained significantly associated with PMPCs. In Model 2, the adjusted ORs per 1 s.d. increase in office SBP and DBP were 0.97 (95% CI: 0.76–1.24) and 1.87 (95% CI: 1.46–2.40), respectively. For HBP, the adjusted ORs per 1 s.d. increase in SBP and DBP were 1.20 (95% CI: 0.92–1.56) and 1.78 (95% CI: 1.38–2.30), respectively. Comparison between MAP and SBP or DBP was not performed because multicollinearity was present. Supplementary Table 8 presents the results of the likelihood ratio test. For both OBP and HBP, adding DBP to a model containing SBP significantly improved model fit (P value < 0.0001 for OBP and P value < 0.0001 for HBP), whereas adding SBP to a model containing DBP did not improve the model fit (P value = 0.8 for OBP and P value = 0.2 for HBP). These findings indicate that, for both OBP and HBP, DBP was more strongly associated with PMPCs than SBP.

### Sensitivity analysis: comparison of associations between OBP and HBP with redefined PMPCs by replacing new-onset HDP with preeclampsia

In sensitivity analyses, PMPCs were redefined by replacing new-onset HDP with preeclampsia. In this analysis, office DBP and MAP, but not SBP, were significantly associated with the redefined PMPCs (Supplementary Fig. 3). Similarly, home DBP and MAP, but not SBP, were significantly associated with higher odds of redefined PMPCs (Supplementary Fig. 4). Although the graded linear association between home SBP and redefined PMPCs was not statistically significant (P values for trend = 0.1), home SBP per 1 s.d increase was associated with a higher risk of PMPCs (Supplementary Fig. 4). When OBP and HBP were included simultaneously in the model, only HBP remained significantly associated with redefined PMPCs across all indices—SBP, DBP, and MAP (Supplementary Fig. 5). Likelihood ratio tests confirmed that adding HBP improved model fit, whereas adding OBP did not (Supplementary Table 9). Details of these sensitivity analyses are provided in the Supplementary Information (Supplementary Figs. 3–6, and Supplementary Tables 9–10).

## Discussion

This prospective cohort study is, to the best of our knowledge, the first to compare maternal HBP and OBP before 20 weeks of gestation in relation to PMPCs risk. Home SBP showed a stronger association with PMPCs than office SBP. Although the difference in DBP was not statistically significant, the effect estimate for HBP was higher than that for OBP. These findings suggest that HBP, particularly SBP, may offer additional predictive value for PMPCs beyond OBP.

To date, only one prior study has compared OBP and HBP before 20 weeks of gestation in relation to infant birth weight, one of several perinatal outcomes, and our findings are largely consistent with that report [18].

Our study adds evidence that HBP during pregnancy may be more closely associated with PMPCs. However, there remains no conclusive evidence that integrating HBP measurement into prenatal care—alongside OBP measurements—improves maternal or neonatal outcomes. Among pregnant women at high risk of preeclampsia, adding HBP measurement to routine prenatal care did not result in earlier clinical diagnosis of HDP or statistically significant improvements in perinatal outcomes [46]. Similarly, in pregnant women with chronic hypertension or gestational hypertension, HBP monitoring during pregnancy did not significantly improve average SBP or alter maternal and neonatal outcomes [47]. Therefore, further research is needed to determine how HBP measurement can best inform perinatal care. For instance, as in nonpregnant adults, it is essential to establish evidence-based thresholds for HBP during pregnancy, taking into account physiological BP changes and their association with perinatal outcomes [48].

The stronger associations observed between home SBP and PMPCs, and the tendency for home DBP and MAP to show stronger associations than office BP in our study, may be partly explained by overestimation of maternal BP because of the white-coat effect and differences in the number of measurements between HBP and OBP. The stronger associations with HBP may also reflect the greater number of data points obtained, which increases the reliability of the estimates. Furthermore, because our HBP data were collected from morning measurements, this may have contributed to the stronger associations observed; morning HBP has been shown to have greater prognostic value for stroke than evening HBP, although the outcome differs from that of our study [49].

In this study, both OBP and HBP demonstrated stronger associations between DBP and PMPCs than between SBP and PMPCs, consistent with our previous findings for infant birth weight [18]. As described in that report, these consistent patterns may be explained by differences in the underlying mechanisms driving elevations in DBP versus SBP [18]. In nonpregnant adults, SBP has been found to reflect arterial compliance, whereas DBP does not [50]. Among young adults, isolated systolic hypertension appears to be driven mainly by elevated stroke volume and increased aortic stiffness. In contrast, elevations in DBP were largely attributable to increased peripheral vascular resistance [51]. After conception, a decline in peripheral vascular resistance, including uteroplacental resistance, typically results in lower DBP during pregnancy [52, 53]. Elevated DBP at 20 weeks of gestation and increased peripheral vascular resistance between 20 and 24 weeks of gestation have been associated with adverse perinatal outcomes. Compared with pregnant women who experienced normal perinatal outcomes, those who developed adverse outcomes had lower stroke volume at 24 weeks of gestation [54]. In a secondary analysis of the Control of Hypertension in Pregnancy Study (CHIPS) trial, higher DBP—but not SBP—was associated with infant birth weight below the 10th percentile and preterm delivery [55]. Collectively, these findings suggest that DBP may be a stronger indicator of perinatal outcome than SBP in pregnant women. Importantly, our analysis revealed that home DBP showed the strongest association with PMPCs among the four indices evaluated (office SBP, office DBP, home SBP, and home DBP), underscoring its potential value as a primary marker for risk stratification.

This study has several strengths. First, the use of both OBP and HBP measurements allowed direct comparison of their predictive value for PMPCs. Second, HBP during pregnancy was assessed using a validated device and measured repeatedly over several days, enhancing measurement reliability. Third, the analysis adjusted for a wide range of maternal characteristics, improving the robustness of the findings. Finally, defining PMPCs as a clinically meaningful composite outcome increases the relevance and applicability of the results to clinical practice.

However, several limitations should be acknowledged. First, this single-center study primarily involved low-risk pregnant women, which may limit generalizability and introduce selection bias. Generalizability to high-risk populations requires confirmation in tertiary care settings. Second, we were unable to examine the association between OBP or HBP and the serum soluble fms-like tyrosine kinase 1 (sFlt-1)/placental growth factor (PlGF) ratio, a well-established predictor of preeclampsia, as it was not measured [56]. Given that gestational hypertension, a subtype of the new-onset HDP, was the most common PMPC, the observed associations may be largely driven by this condition. Nonetheless, in sensitivity analyses redefining PMPCs by replacing new-onset HDP with preeclampsia, only HBP indices—SBP, DBP, and MAP—remained significantly associated when modeled with OBP. These results underscore the robustness and predictive value of HBP across outcome definitions.

In conclusion, home SBP was more strongly associated with PMPCs than office SBP. Although home DBP was not statistically stronger than office DBP, its effect estimate was higher. These findings support the added value of HBP monitoring during pregnancy for predicting PMPCs.

## Supplementary information

Supplementary Information
