# Supplementary material for: Comparison of the associations between office and home BP with placenta-mediated pregnancy complications: the BOSHI study
Source: Hypertens Res. 2025 Nov 20;49(3):926–37. doi: 10.1038/s41440-025-02439-x (PMC12960211; doi:10.1038/s41440-025-02439-x)
Supplement: Supplementary file 1 — Supplementary Information [file 41440_2025_2439_MOESM1_ESM.docx]

**SUPPLEMENTARY INFORMATION**

**Other variables used in this study**

Information on maternal age, parity, last menstrual period, estimated date of confinement (determined based on fetal crown-rump length measured by ultrasound before 12 weeks of gestation), gestational age at each prenatal visit, maternal body weight at each prenatal visit, and medical history was extracted from obstetric medical records. In addition, data on pre-pregnancy weight and height, smoking history (no smoking before conception, smoking until conception was recognized, or smoking during pregnancy), alcohol consumption history (no alcohol intake before conception, alcohol intake until conception was recognized, or alcohol intake during pregnancy), and family history of hypertension were collected through self-administered questionnaires and interviews conducted by midwives. Pre-pregnancy body mass index (BMI) (kg/m²) was calculated using the standard formula: pre-pregnancy body weight in kilograms divided by height in meters squared. Pre-pregnancy BMI was classified into underweight (<18.5 kg/m²), normal weight (18.5–24.9 kg/m²), and overweight/obese (≥25.0 kg/m²). HbA1c levels before 20 weeks of gestation were measured as previously described. [1] The season of the estimated date of confinement was categorized as spring (March-May), summer (June-August), autumn (September-November), or winter (December-February).

**Sensitivity Analysis: Comparison of associations between office blood pressure (OBP) and home blood pressure (HBP) with redefined placenta-mediated pregnancy complications (PMPCs), in which new-onset hypertensive disorders of pregnancy (HDP) was replaced by preeclampsia**

Supplementary Figure 3 illustrates the association between OBP with the redefined PMPCs, in which new-onset HDP was replaced by preeclampsia. Office systolic blood pressure (SBP) was not statistically significantly associated with the redefined PMPCs; the adjusted odds ratio (OR) per 1s.d. increase in office SBP was 1.04 (95% confidence interval [CI]: 0.83–1.29) in Model 2. In contrast, higher levels of office diastolic blood pressure (DBP) and mean arterial pressure (MAP) were significantly associated with increased odds of the redefined PMPCs (P-values for trend = 0.006 and 0.03, respectively) in Model 2.

Supplementary Figure 4 also illustrates the association between HBP with the redefined PMPCs. Although a linear graded association between home SBP and the redefined PMPCs was not statistically significant (P-values for trend = 0.1), the adjusted OR per 1s.d. increase in home SBP was 1.35 (95% CI: 1.08–1.69) in Model 2. Both home DBP and MAP were significantly associated with increasing odds of the redefined PMPCs, with adjusted ORs per 1s.d. increase were 1.47 (95% CI: 1.18–1.82) and 1.46 (95% CI: 1.18–1.82), respectively, in Model 2.

Supplementary Figure 5 shows a comparison of the associations between OBP and HBP before 20 weeks of gestation with redefined PMPCs. When both OBP and HBP were included simultaneously in the model as continuous variables per 1s.d. increase, only HBP showed a statistically significant association with the redefined PMPCs for all three indices—SBP, DBP, and MAP. As shown in Supplementary Table 9, the likelihood ratio test indicated that adding HBP to a model including OBP significantly improved model fit for all three indices—SBP (P-value = 0.004), DBP (P-value = 0.01), and MAP (P-value = 0.03) in Model 2. In contrast, adding OBP to a model that already included HBP did not result in a statistically significant improvement in model fit for all three indices—SBP (P-value = 0.2), DBP (P-value = 0.5), and MAP (P-value = 0.8) in Model 2.

As shown in Supplementary Figure 6, when both SBP and DBP were simultaneously included in the model as continuous variables per 1 s.d. increase, only DBP was statistically significantly associated with the development of the redefined PMPCs in both OBP and HBP. Furthermore, as presented in Supplementary Table 10, the likelihood ratio tests demonstrated that, in Model 2, adding DBP to a model that included SBP significantly improved model fit (P-value = 0.002 for OBP and 0.02 for HBP), whereas adding SBP to a model that included DBP did not improve the fit in both OBP and HBP (P-value = 0.06 for OBP and 0.9 for HBP).

**Members of the BOSHI Study Group**

Medical members (Obstetrics): Noriyuki Iwama, Hidekazu Nishigori, Kohei Tanaka, Takashi Sugiyama, Junichi Sugawara, Nobuo Yaegashi, Kazuhiko Hoshi, Kunihiko Okamura, Masakuni Suzuki. Medical members (Internal Medicine): Hirohito Metoki, Kei Asayama, Ryusuke Inoue, Masahiro Kikuya, Takayoshi Ohkubo, Shinichi Kuriyama, Yutaka Imai.

Coordinating members: Taku Obara, Mami Ishikuro, Rie Tsuchida, Azusa Hara, Takuo Hirose, Takeshi Kobayashi, Kenta Gonokami, Takanao Hashimoto, Yumiko Watanabe, Misato Nishimura, Maiko Kawaguchi, Yurie Sato, Minako Hoshikawa, Ayano Sasaki, Kasumi Sakurai, Michihiro Sato, Konomi Akutsu, Mami Yamamoto, Aya shiraishi, Miki Hosaka. clinical examination members: Ikuo Tachibana, Maki Omura, Mikiko Ishikawa, Yoshimi Fujii, Hidemi Kobayashi, Kazuyuki Akaishi. Pharmaceutical members: Yuko Kikuchi, Kei Tate, Chieko Koishi, Saori Sugawara. Recruitment members: Katsuyo Yagihashi, Junko Saitou, Hiromi Sasaki, Tomoko Suzuki, Junko Takahashi, Yoko Narita, Satoko Shigihara, Hideko Tada, Yumi Hamada. Outpatient management members: Nozomi Satou. Nami Satou, Setsuko Sai, Nana Atsumi, Naoko Sekine, Yukari Ueno, Yu Itou. Inpatient management members: Yukie Obara, Nami Onodera, Asako Sato, Youko Iwasa, Mamiko Abe, Yukari Kido, Risako Komuro, Yukiko Nakamura, Marie Watanabe, Chikako Matsumoto, Koto Oyama, Aya Takahashi, Michiko Kojima, Miyuki Abe, Mariko Sane, Mana Takahashi, Kana Sugata Miho Igari, Haruhi Sasaki, Mizuki Kobayashi, Aya Kikuchi, Risa Yamamoto, Akiho Goto, Eri Yamauchi, Mika Chiba, Sakiko Ota, Hiromi Ishikawa, Akemi Sasaki, Tomoko Kawamura, Hiroko Hiji, Misaki Kishinami, Yurie Kowata, Eiko Yamauchi, Yasuko Takahashi, Naho Sato.

**References**

1. Izumi S, Iwama N, Hamada H, Obara T, Ishikuro M, Satoh M, et al. Associations of fasting plasma glucose and glycosylated hemoglobin levels at less than 24 weeks of gestation with hypertensive disorders of pregnancy: the BOSHI study. Endocr J. 2024;71:979-93.

**Supplementary Table 1. Number and proportion of participants according to the number of office BP and home BP measurements**

| **Number of BP measurements** | **All participants (N=975)** |
| --- | --- |
| **Number of office BP measurements per antenatal visit, N (%)** |  |
| Once | 76 (7.8) |
| Twice | 899 (92.2) |
| **Number of home BP measurements in one week, N (%)** |  |
| Once | 281 (28.8) |
| Twice | 202 (20.7) |
| 3 times | 171 (17.5) |
| 4 times | 118 (12.1) |
| 5 times | 71 (7.3) |
| 6 times | 73 (7.5) |
| 7 times | 59 (6.1) |

Abbreviations: BP, blood pressure.

**Supplementary Table 2. Characteristics according to quartiles of office SBP**

| **Variables** | **Participants according to quartiles of office SBP** | | | |
| --- | --- | --- | --- | --- |
|  | **Quartile 1 (81.0－100.5 mmHg), N=240** | **Quartile 2 (101.0－107.0 mmHg), N=253** | **Quartile 3 (107.5－114.5 mmHg), N=239** | **Quartile 4 (115.0－137.0 mmHg), N=243** |
| **Maternal age, years** | 31.6 (4.7) | 31.2 (4.6) | 31.1 (4.7) | 31.1 (5.0) |
| <35 years, N (%) | 174 (72.5) | 184 (72.7) | 184 (77.0) | 174 (71.6) |
| ≥35 years, N (%) | 66 (27.5) | 68 (26.9) | 54 (22.6) | 69 (28.4) |
| Missing, N (%) | 0 (0.0) | 1 (0.4) | 1 (0.4) | 0 (0.0) |
| **Height, cm** | 158.5 (5.1) | 158.4 (5.5) | 158.5 (5.1) | 158.7 (5.0) |
| **Pre-pregnancy body weight, kg** | 51.4 (6.4) | 53.3 (7.2) | 55.2 (8.9) | 56.2 (10.3) |
| **Pre-pregnancy BMI, kg/m²** | 20.4 (2.2) | 21.2 (2.7) | 22.0 (3.3) | 22.3 (3.8) |
| Underweight (<18.5 kg/m²), N (%) | 42 (17.5) | 32 (12.6) | 26 (10.9) | 24 (9.9) |
| Normal weight (18.5－24.9 kg/m²), N (%) | 189 (78.8) | 197 (77.9) | 177 (74.1) | 172 (70.8) |
| Overweight/Obese (≥25.0 kg/m²), N (%) | 9 (3.8) | 24 (9.5) | 36 (15.1) | 47 (19.3) |
| **Parity, N (%)** |  |  |  |  |
| Primipara | 134 (55.8) | 147 (58.1) | 149 (62.3) | 157 (64.6) |
| Multipara without HDP in a previous pregnancy | 101 (42.1) | 98 (38.7) | 77 (32.2) | 80 (32.9) |
| Multipara with HDP in a previous pregnancy | 2 (0.8) | 5 (2.0) | 7 (2.9) | 6 (2.5) |
| Missing | 3 (1.3) | 3 (1.2) | 6 (2.5) | 0 (0.0) |
| **ART, N (%)** | 7 (2.9) | 2 (0.8) | 9 (3.8) | 9 (3.7) |
| **Family history of hypertension, N (%)** | 10 (4.2) | 18 (7.1) | 10 (4.2) | 12 (4.9) |
| **Smoking status, N (%)** |  |  |  |  |
| No smoking before conception | 212 (88.3) | 214 (84.6) | 196 (82.0) | 202 (83.1) |
| Until conception was recognized | 20 (8.3) | 28 (11.1) | 38 (15.9) | 34 (14.0) |
| Smoking during pregnancy | 8 (3.3) | 11 (4.3) | 5 (2.1) | 7 (2.9) |
| **Alcohol drinking, N (%)** |  |  |  |  |
| No alcohol intake before conception | 119 (49.6) | 131 (51.8) | 126 (52.7) | 142 (58.4) |
| Until conception was recognized | 97 (40.4) | 94 (37.2) | 85 (35.6) | 73 (30.0) |
| Alcohol intake during pregnancy | 20 (8.3) | 27 (10.7) | 26 (10.9) | 26 (10.7) |
| Missing | 4 (1.7) | 1 (0.4) | 2 (0.8) | 2 (0.8) |
| **HbA1c before 20 weeks of gestation, %** | 5.0 (0.2) | 5.0 (0.3) | 5.0 (0.2) | 5.0 (0.2) |
| **Season of estimated date of confinement, N (%)** |  |  |  |  |
| Spring | 63 (26.3) | 58 (22.9) | 61 (25.5) | 60 (24.7) |
| Summer | 45 (18.8) | 56 (22.1) | 64 (26.8) | 81 (33.3) |
| Autumn | 67 (27.9) | 65 (25.7) | 61 (25.5) | 59 (24.3) |
| Winter | 65 (27.1) | 74 (29.2) | 53 (22.2) | 43 (17.7) |
| **Hyperthyroidism, N (%)** | 1 (0.4) | 2 (0.8) | 2 (0.8) | 2 (0.8) |
| **Hypothyroidism, N (%)** | 0 (0.0) | 1 (0.4) | 1 (0.4) | 0 (0.0) |
| **Chronic kidney disease, N (%)** | 0 (0.0) | 0 (0.0) | 0 (0.0) | 0 (0.0) |
| **Gestational age when office BP was measured, weeks** | 14.0 (2.2) | 13.9 (2.3) | 13.8 (2.3) | 13.8 (2.4) |
| **Gestational age when home BP was measured, weeks** | 14.2 (2.2) | 14.0 (2.2) | 14.0 (2.2) | 13.9 (2.3) |
| **Number of office BP measurements, median (range)** | 2 (1―2) | 2 (1―2) | 2 (1―2) | 2 (1―2) |
| **Number of home BP measurements, median (range)** | 2 (1―7) | 3 (1―7) | 3 (1―7) | 3 (1―7) |
| **Office SBP before 20 weeks of gestation, mmHg** | 95 (4) | 104 (2) | 111 (2) | 122 (5) |
| **Office DBP before 20 weeks of gestation, mmHg** | 59 (5) | 64 (6) | 67 (6) | 74 (7) |
| **Office MAP before 20 weeks of gestation, mmHg** | 71 (4) | 77 (4) | 82 (4) | 90 (5) |
| **Home SBP before 20 weeks of gestation, mmHg** | 98 (7) | 102 (7) | 105 (8) | 111 (8) |
| **Home DBP before 20 weeks of gestation, mmHg** | 59 (6) | 61 (6) | 63 (6) | 67 (7) |
| **Home MAP before 20 weeks of gestation, mmHg** | 72 (6) | 75 (6) | 77 (6) | 82 (7) |
| **PMPCs, N (%)** | 28 (11.7) | 37 (14.6) | 39 (16.3) | 66 (27.2) |
| New-onset HDP, N (%) | 9 (3.8) | 14 (5.5) | 23 (9.6) | 46 (18.9) |
| Preeclampsia, N (%) | 2 (0.8) | 2 (0.8) | 7 (2.9) | 6 (2.5) |
| Gestational hypertension, N (%) | 7 (2.9) | 12 (4.7) | 16 (6.7) | 40 (16.5) |
| Placental abruption, N (%) | 1 (0.4) | 0 (0.0) | 1 (0.4) | 1 (0.4) |
| Preterm delivery (<37 weeks of gestation), N (%) | 4 (1.7) | 7 (2.8) | 9 (3.8) | 9 (3.7) |
| SGA infants (Birth weight <10th percentile), N (%) | 14 (5.8) | 21 (8.3) | 13 (5.4) | 17 (7.0) |
| Stillbirth at ≥28 weeks of gestation, N (%) | 0 (0.0) | 2 (0.8) | 0 (0.0) | 0 (0.0) |
| **Redefined PMPCs by replacing new-onset HDP with preeclampsia, N (%)** | 21 (8.8) | 28 (11.1) | 26 (10.9) | 28 (11.5) |
| **Infant sex, N (%)** |  |  |  |  |
| Male | 123 (51.3) | 144 (56.9) | 117 (49.0) | 123 (50.6) |
| Female | 117 (48.8) | 109 (43.1) | 122 (51.0) | 120 (49.4) |
| **Delivery week, weeks** | 39.8 (1.2) | 39.5 (1.4) | 39.6 (1.8) | 39.4 (2.2) |
| **Infant birth weight, g** | 3,095 (376) | 3,031 (397) | 3,061 (436) | 3,029 (467) |
| Low birth weight (<2,500 g), N (%) | 13 (5.4) | 21 (8.3) | 18 (7.5) | 18 (7.4) |

PMPCs include at least one of the following perinatal outcomes: new-onset HDP, placental abruption, preterm delivery, SGA infants, and stillbirth at ≥28 weeks of gestation.

Redefined PMPCs include at least one of the following perinatal outcomes: preeclampsia, placental abruption, preterm delivery, SGA infants, and stillbirth at ≥28 weeks of gestation.

Abbreviations: ART, assisted reproductive technology; BMI, body mass index; BP, blood pressure; DBP, diastolic blood pressure; HDP, hypertensive disorders of pregnancy; MAP, mean arterial pressure; PMPCs, placenta-mediated pregnancy complications; SBP, systolic blood pressure; s.d., standard deviation; SGA, small for gestational age.

**Supplementary Table 3. Characteristics according to quartiles of office DBP**

| **Variables** | **Participants according to quartiles of Office DBP** | | | |
| --- | --- | --- | --- | --- |
|  | **Quartile 1 (40.0－59.5 mmHg), N=237** | **Quartile 2 (60.0－65.0 mmHg), N=244** | **Quartile 3 (65.5－71.5 mmHg), N=256** | **Quartile 4 (72.0－89.5 mmHg), N=238** |
| **Maternal age, years** | 31.2 (4.7) | 31.2 (4.5) | 30.9 (4.8) | 31.6 (4.9) |
| <35 years, N (%) | 170 (71.7) | 186 (76.2) | 197 (77.0) | 163 (68.5) |
| ≥35 years, N (%) | 67 (28.3) | 57 (23.4) | 58 (22.7) | 75 (31.5) |
| Missing, N (%) | 0 (0.0) | 1 (0.4) | 1 (0.4) | 0 (0.0) |
| **Height, cm** | 158.7 (5.0) | 158.3 (4.9) | 158.4 (5.7) | 158.6 (5.1) |
| **Pre-pregnancy body weight, kg** | 52.4 (6.8) | 53.3 (7.8) | 53.8 (8.1) | 56.6 (10.4) |
| **Pre-pregnancy BMI, kg/m²** | 20.8 (2.4) | 21.2 (2.8) | 21.4 (3.0) | 22.5 (4.0) |
| Underweight (<18.5 kg/m²), N (%) | 32 (13.5) | 28 (11.5) | 37 (14.5) | 27 (11.3) |
| Normal weight (18.5－24.9 kg/m²), N (%) | 194 (81.9) | 194 (79.5) | 190 (74.2) | 157 (66.0) |
| Overweight/Obese (≥25.0 kg/m²), N (%) | 11 (4.6) | 22 (9.0) | 29 (11.3) | 54 (22.7) |
| **Parity, N (%)** |  |  |  |  |
| Primipara | 131 (55.3) | 134 (54.9) | 170 (66.4) | 152 (63.9) |
| Multipara without HDP in a previous pregnancy | 104 (43.9) | 97 (39.8) | 78 (30.5) | 77 (32.4) |
| Multipara with HDP in a previous pregnancy | 2 (0.8) | 7 (2.9) | 3 (1.2) | 8 (3.4) |
| Missing | 0 (0.0) | 6 (2.5) | 5 (2.0) | 1 (0.4) |
| **ART, N (%)** | 4 (1.7) | 4 (1.6) | 8 (3.1) | 11 (4.6) |
| **Family history of hypertension, N (%)** | 13 (5.5) | 13 (5.3) | 13 (5.1) | 11 (4.6) |
| **Smoking status, N (%)** |  |  |  |  |
| No smoking before conception | 199 (84.0) | 205 (84.0) | 211 (82.4) | 209 (87.8) |
| Until conception was recognized | 28 (11.8) | 33 (13.5) | 34 (13.3) | 25 (10.5) |
| Smoking during pregnancy | 10 (4.2) | 6 (2.5) | 11 (4.3) | 4 (1.7) |
| **Alcohol drinking, N (%)** |  |  |  |  |
| No alcohol intake before conception | 117 (49.4) | 122 (50.0) | 142 (55.5) | 137 (57.6) |
| Until conception was recognized | 91 (38.4) | 97 (39.8) | 86 (33.6) | 75 (31.5) |
| Alcohol intake during pregnancy | 23 (9.7) | 24 (9.8) | 26 (10.2) | 26 (10.9) |
| Missing | 6 (2.5) | 1 (0.4) | 2 (0.8) | 0 (0.0) |
| **HbA1c before 20 weeks of gestation, %** | 5.0 (0.3) | 5.0 (0.3) | 5.0 (0.2) | 5.1 (0.2) |
| **Season of estimated date of confinement, N (%)** |  |  |  |  |
| Spring | 50 (21.1) | 62 (25.4) | 64 (25.0) | 66 (27.7) |
| Summer | 58 (24.5) | 69 (28.3) | 53 (20.7) | 66 (27.7) |
| Autumn | 60 (25.3) | 61 (25.0) | 72 (28.1) | 59 (24.8) |
| Winter | 69 (29.1) | 52 (21.3) | 67 (26.2) | 47 (19.7) |
| **Hyperthyroidism, N (%)** | 1 (0.4) | 2 (0.8) | 3 (1.2) | 1 (0.4) |
| **Hypothyroidism, N (%)** | 1 (0.4) | 1 (0.4) | 0 (0.0) | 0 (0.0) |
| **Chronic kidney disease, N (%)** | 0 (0.0) | 0 (0.0) | 0 (0.0) | 0 (0.0) |
| **Gestational age when office BP was measured, weeks** | 14.5 (2.4) | 14.0 (2.4) | 13.7 (2.2) | 13.3 (2.1) |
| **Gestational age when home BP was measured, weeks** | 14.6 (2.2) | 14.2 (2.3) | 13.9 (2.1) | 13.6 (2.1) |
| **Number of office BP measurements, median (range)** | 2 (1―2) | 2 (1―2) | 2 (1―2) | 2 (1―2) |
| **Number of home BP measurements, median (range)** | 2 (1―7) | 3 (1―7) | 3 (1―7) | 3 (1―7) |
| **Office SBP before 20 weeks of gestation, mmHg** | 100 (8) | 105 (8) | 110 (8) | 118 (8) |
| **Office DBP before 20 weeks of gestation, mmHg** | 56 (3) | 63 (2) | 69 (2) | 77 (4) |
| **Office MAP before 20 weeks of gestation, mmHg** | 70 (4) | 77 (3) | 82 (3) | 91 (5) |
| **Home SBP before 20 weeks of gestation, mmHg** | 100 (8) | 102 (8) | 105 (8) | 110 (9) |
| **Home DBP before 20 weeks of gestation, mmHg** | 58 (6) | 61 (5) | 64 (6) | 68 (7) |
| **Home MAP before 20 weeks of gestation, mmHg** | 72 (6) | 75 (5) | 77 (6) | 82 (7) |
| **PMPCs, N (%)** | 17 (7.2) | 32 (13.1) | 48 (18.8) | 73 (30.7) |
| New-onset HDP, N (%) | 4 (1.7) | 11 (4.5) | 25 (9.8) | 52 (21.8) |
| Preeclampsia, N (%) | 1 (0.4) | 1 (0.4) | 6 (2.3) | 9 (3.8) |
| Gestational hypertension, N (%) | 3 (1.3) | 10 (4.1) | 19 (7.4) | 43 (18.1) |
| Placental abruption, N (%) | 0 (0.0) | 2 (0.8) | 1 (0.4) | 0 (0.0) |
| Preterm delivery (<37 weeks of gestation), N (%) | 3 (1.3) | 7 (2.9) | 8 (3.1) | 11 (4.6) |
| SGA infants (Birth weight <10th percentile), N (%) | 11 (4.6) | 16 (6.6) | 20 (7.8) | 18 (7.6) |
| Stillbirth at ≥28 weeks of gestation, N (%) | 0 (0.0) | 2 (0.8) | 0 (0.0) | 0 (0.0) |
| **Redefined PMPCs by replacing new-onset HDP with preeclampsia, N (%)** | 14 (5.9) | 24 (9.8) | 33 (12.9) | 32 (13.4) |
| **Infant sex, N (%)** |  |  |  |  |
| Male | 111 (46.8) | 139 (57.0) | 134 (52.3) | 123 (51.7) |
| Female | 126 (53.2) | 105 (43.0) | 122 (47.7) | 115 (48.3) |
| **Delivery week, weeks** | 39.8 (1.1) | 39.6 (1.4) | 39.6 (1.9) | 39.4 (2.1) |
| **Infant birth weight, g** | 3,110 (363) | 3,064 (411) | 3,021 (436) | 3,021 (460) |
| Low birth weight (<2,500 g), N (%) | 14 (5.9) | 18 (7.4) | 15 (5.9) | 23 (9.7) |

PMPCs include at least one of the following perinatal outcomes: new-onset HDP, placental abruption, preterm delivery, SGA infants, and stillbirth at ≥28 weeks of gestation.

Redefined PMPCs include at least one of the following perinatal outcomes: preeclampsia, placental abruption, preterm delivery, SGA infants, and stillbirth at ≥28 weeks of gestation.

Abbreviations: ART, assisted reproductive technology; BMI, body mass index; BP, blood pressure; DBP, diastolic blood pressure; HDP, hypertensive disorders of pregnancy; MAP, mean arterial pressure; PMPCs, placenta-mediated pregnancy complications; SBP, systolic blood pressure; s.d., standard deviation; SGA, small for gestational age.

**Supplementary Table 4. Characteristics according to quartiles of office MAP**

| **Variables** | **Participants according to quartiles of office MAP** | | | |
| --- | --- | --- | --- | --- |
|  | **Quartile 1 (53.7－73.8 mmHg), N=247** | **Quartile 2 (74.0－79.3 mmHg), N=238** | **Quartile 3 (79.5－85.3 mmHg), N=243** | **Quartile 4 (85.5－103.7 mmHg), N=247** |
| **Maternal age, years** | 31.6 (4.6) | 30.9 (4.7) | 31.3 (4.6) | 31.3 (5.1) |
| <35 years, N (%) | 177 (71.7) | 183 (76.9) | 183 (75.3) | 173 (70.0) |
| ≥35 years, N (%) | 70 (28.3) | 54 (22.7) | 59 (24.3) | 74 (30.0) |
| Missing, N (%) | 0 (0.0) | 1 (0.4) | 1 (0.4) | 0 (0.0) |
| **Height, cm** | 158.4 (4.8) | 158.6 (5.2) | 158.5 (5.7) | 158.5 (5.0) |
| **Pre-pregnancy body weight, kg** | 51.7 (6.3) | 53.5 (7.8) | 54.7 (8.1) | 56.2 (10.7) |
| **Pre-pregnancy BMI, kg/m²** | 20.6 (2.2) | 21.2 (2.7) | 21.7 (3.0) | 22.4 (4.0) |
| Underweight (<18.5 kg/m²), N (%) | 36 (14.6) | 28 (11.8) | 29 (11.9) | 31 (12.6) |
| Normal weight (18.5－24.9 kg/m²), N (%) | 201 (81.4) | 189 (79.4) | 182 (74.9) | 163 (66.0) |
| Overweight/Obese (≥25.0 kg/m²), N (%) | 10 (4.0) | 21 (8.8) | 32 (13.2) | 53 (21.5) |
| **Parity, N (%)** |  |  |  |  |
| Primipara | 132 (53.4) | 135 (56.7) | 159 (65.4) | 161 (65.2) |
| Multipara without HDP in a previous pregnancy | 112 (45.3) | 91 (38.2) | 75 (30.9) | 78 (31.6) |
| Multipara with HDP in a previous pregnancy | 2 (0.8) | 7 (2.9) | 3 (1.2) | 8 (3.2) |
| Missing | 1 (0.4) | 5 (2.1) | 6 (2.5) | 0 (0.0) |
| **ART, N (%)** | 4 (1.6) | 5 (2.1) | 8 (3.3) | 10 (4.0) |
| **Family history of hypertension, N (%)** | 14 (5.7) | 7 (2.9) | 20 (8.2) | 9 (3.6) |
| **Smoking status, N (%)** |  |  |  |  |
| No smoking before conception | 214 (86.6) | 197 (82.8) | 201 (82.7) | 212 (85.8) |
| Until conception was recognized | 24 (9.7) | 31 (13.0) | 36 (14.8) | 29 (11.7) |
| Smoking during pregnancy | 9 (3.6) | 10 (4.2) | 6 (2.5) | 6 (2.4) |
| **Alcohol drinking, N (%)** |  |  |  |  |
| No alcohol intake before conception | 127 (51.4) | 123 (51.7) | 123 (50.6) | 145 (58.7) |
| Until conception was recognized | 91 (36.8) | 93 (39.1) | 92 (37.9) | 73 (29.6) |
| Alcohol intake during pregnancy | 25 (10.1) | 19 (8.0) | 27 (11.1) | 28 (11.3) |
| Missing | 4 (1.6) | 3 (1.3) | 1 (0.4) | 1 (0.4) |
| **HbA1c before 20 weeks of gestation, %** | 5.0 (0.3) | 5.0 (0.3) | 5.0 (0.2) | 5.1 (0.2) |
| **Season of estimated date of confinement, N (%)** |  |  |  |  |
| Spring | 57 (23.1) | 56 (23.5) | 61 (25.1) | 68 (27.5) |
| Summer | 54 (21.9) | 66 (27.7) | 54 (22.2) | 72 (29.1) |
| Autumn | 65 (26.3) | 59 (24.8) | 69 (28.4) | 59 (23.9) |
| Winter | 71 (28.7) | 57 (23.9) | 59 (24.3) | 48 (19.4) |
| **Hyperthyroidism, N (%)** | 1 (0.4) | 1 (0.4) | 4 (1.6) | 1 (0.4) |
| **Hypothyroidism, N (%)** | 1 (0.4) | 0 (0.0) | 1 (0.4) | 0 (0.0) |
| **Chronic kidney disease, N (%)** | 0 (0.0) | 0 (0.0) | 0 (0.0) | 0 (0.0) |
| **Gestational age when office BP was measured, weeks** | 14.3 (2.3) | 14.0 (2.4) | 13.6 (2.1) | 13.5 (2.2) |
| **Gestational age when home BP was measured, weeks** | 14.5 (2.2) | 14.2 (2.4) | 13.8 (2.0) | 13.7 (2.2) |
| **Number of office BP measurements, median (range)** | 2 (1―2) | 2 (1―2) | 2 (1―2) | 2 (1―2) |
| **Number of home BP measurements, median (range)** | 2 (1―7) | 2 (1―7) | 3 (1―7) | 3 (1―7) |
| **Office SBP before 20 weeks of gestation, mmHg** | 97 (5) | 105 (5) | 110 (5) | 120 (7) |
| **Office DBP before 20 weeks of gestation, mmHg** | 56 (4) | 63 (3) | 68 (3) | 76 (5) |
| **Office MAP before 20 weeks of gestation, mmHg** | 70 (3) | 77 (2) | 82 (2) | 91 (4) |
| **Home SBP before 20 weeks of gestation, mmHg** | 99 (7) | 103 (7) | 104 (8) | 110 (9) |
| **Home DBP before 20 weeks of gestation, mmHg** | 58 (6) | 61 (5) | 63 (6) | 68 (6) |
| **Home MAP before 20 weeks of gestation, mmHg** | 72 (5) | 75 (5) | 77 (6) | 82 (7) |
| **PMPCs, N (%)** | 24 (9.7) | 29 (12.2) | 42 (17.3) | 75 (30.4) |
| New-onset HDP, N (%) | 7 (2.8) | 9 (3.8) | 24 (9.9) | 52 (21.1) |
| Preeclampsia, N (%) | 1 (0.4) | 1 (0.4) | 6 (2.5) | 9 (3.6) |
| Gestational hypertension, N (%) | 6 (2.4) | 8 (3.4) | 18 (7.4) | 43 (17.4) |
| Placental abruption, N (%) | 1 (0.4) | 1 (0.4) | 1 (0.4) | 0 (0.0) |
| Preterm delivery (<37 weeks of gestation), N (%) | 3 (1.2) | 8 (3.4) | 7 (2.9) | 11 (4.5) |
| SGA infants (Birth weight <10th percentile), N (%) | 14 (5.7) | 14 (5.9) | 17 (7.0) | 20 (8.1) |
| Stillbirth at ≥28 weeks of gestation, N (%) | 0 (0.0) | 2 (0.8) | 0 (0.0) | 0 (0.0) |
| **Redefined PMPCs by replacing new-onset HDP with preeclampsia, N (%)** | 18 (7.3) | 23 (9.7) | 29 (11.9) | 33 (13.4) |
| **Infant sex, N (%)** |  |  |  |  |
| Male | 120 (48.6) | 138 (58.0) | 123 (50.6) | 126 (51.0) |
| Female | 127 (51.4) | 100 (42.0) | 120 (49.4) | 121 (49.0) |
| **Delivery week, weeks** | 39.8 (1.2) | 39.6 (1.4) | 39.5 (1.7) | 39.4 (2.2) |
| **Infant birth weight, g** | 3,092 (363) | 3,081 (411) | 3,030 (420) | 3,011 (476) |
| Low birth weight (<2,500 g), N (%) | 14 (5.7) | 18 (7.6) | 15 (6.2) | 23 (9.3) |

PMPCs include at least one of the following perinatal outcomes: new-onset HDP, placental abruption, preterm delivery, SGA infants, and stillbirth at ≥28 weeks of gestation.

Redefined PMPCs include at least one of the following perinatal outcomes: preeclampsia, placental abruption, preterm delivery, SGA infants, and stillbirth at ≥28 weeks of gestation.

Abbreviations: ART, assisted reproductive technology; BMI, body mass index; BP, blood pressure; DBP, diastolic blood pressure; HDP, hypertensive disorders of pregnancy; MAP, mean arterial pressure; PMPCs, placenta-mediated pregnancy complications; SBP, systolic blood pressure; s.d., standard deviation; SGA, small for gestational age.

**Supplementary Table 5. Characteristics according to quartiles of home SBP**

| **Variables** | **Participants according to quartiles of home SBP** | | | |
| --- | --- | --- | --- | --- |
|  | **Quartile 1 (78.5－98.0 mmHg), N=252** | **Quartile 2 (98.2－103.5 mmHg), N=238** | **Quartile 3 (103.7－109.8 mmHg), N=241** | **Quartile 4 (110.0－132.0 mmHg), N=244** |
| **Maternal age, years** | 31.2 (4.3) | 30.9 (4.8) | 31.6 (4.8) | 31.4 (5.1) |
| <35 years, N (%) | 195 (77.4) | 178 (74.8) | 167 (69.3) | 176 (72.1) |
| ≥35 years, N (%) | 57 (22.6) | 59 (24.8) | 73 (30.3) | 68 (27.9) |
| Missing, N (%) | 0 (0.0) | 1 (0.4) | 1 (0.4) | 0 (0.0) |
| **Height, cm** | 158.7 (5.1) | 158.0 (5.4) | 158.4 (4.9) | 158.9 (5.3) |
| **Pre-pregnancy body weight, kg** | 51.1 (6.1) | 53.0 (7.4) | 54.5 (8.3) | 57.5 (10.5) |
| **Pre-pregnancy BMI, kg/m²** | 20.3 (2.1) | 21.2 (2.7) | 21.7 (3.1) | 22.8 (3.8) |
| Underweight (<18.5 kg/m²), N (%) | 50 (19.8) | 27 (11.3) | 27 (11.2) | 20 (8.2) |
| Normal weight (18.5－24.9 kg/m²), N (%) | 196 (77.8) | 189 (79.4) | 183 (75.9) | 167 (68.4) |
| Overweight/Obese (≥25.0 kg/m²), N (%) | 6 (2.4) | 22 (9.2) | 31 (12.9) | 57 (23.4) |
| **Parity, N (%)** |  |  |  |  |
| Primipara | 158 (62.7) | 151 (63.4) | 139 (57.7) | 139 (57.0) |
| Multipara without HDP in a previous pregnancy | 90 (35.7) | 83 (34.9) | 91 (37.8) | 92 (37.7) |
| Multipara with HDP in a previous pregnancy | 2 (0.8) | 3 (1.3) | 6 (2.5) | 9 (3.7) |
| Missing | 2 (0.8) | 1 (0.4) | 5 (2.1) | 4 (1.6) |
| **ART, N (%)** | 4 (1.6) | 4 (1.7) | 9 (3.7) | 10 (4.1) |
| **Family history of hypertension, N (%)** | 14 (5.6) | 8 (3.4) | 14 (5.8) | 14 (5.7) |
| **Smoking status, N (%)** |  |  |  |  |
| No smoking before conception | 227 (90.1) | 194 (81.5) | 205 (85.1) | 198 (81.1) |
| Until conception was recognized | 20 (7.9) | 37 (15.5) | 31 (12.9) | 32 (13.1) |
| Smoking during pregnancy | 5 (2.0) | 7 (2.9) | 5 (2.1) | 14 (5.7) |
| **Alcohol drinking, N (%)** |  |  |  |  |
| No alcohol intake before conception | 124 (49.2) | 112 (47.1) | 137 (56.8) | 145 (59.4) |
| Until conception was recognized | 97 (38.5) | 97 (40.8) | 79 (32.8) | 76 (31.1) |
| Alcohol intake during pregnancy | 28 (11.1) | 25 (10.5) | 24 (10.0) | 22 (9.0) |
| Missing | 3 (1.2) | 4 (1.7) | 1 (0.4) | 1 (0.4) |
| **HbA1c before 20 weeks of gestation, %** | 5.0 (0.2) | 5.0 (0.2) | 5.0 (0.2) | 5.0 (0.3) |
| **Season of estimated date of confinement, N (%)** |  |  |  |  |
| Spring | 61 (24.2) | 57 (23.9) | 59 (24.5) | 65 (26.6) |
| Summer | 43 (17.1) | 55 (23.1) | 65 (27.0) | 83 (34.0) |
| Autumn | 53 (21.0) | 69 (29.0) | 73 (30.3) | 57 (23.4) |
| Winter | 95 (37.7) | 57 (23.9) | 44 (18.3) | 39 (16.0) |
| **Hyperthyroidism, N (%)** | 3 (1.2) | 1 (0.4) | 0 (0.0) | 3 (1.2) |
| **Hypothyroidism, N (%)** | 0 (0.0) | 0 (0.0) | 2 (0.8) | 0 (0.0) |
| **Chronic kidney disease, N (%)** | 0 (0.0) | 0 (0.0) | 0 (0.0) | 0 (0.0) |
| **Gestational age when Office BP was measured, weeks** | 14.0 (2.3) | 13.7 (2.2) | 14.0 (2.4) | 13.8 (2.3) |
| **Gestational age when home BP was measured, weeks** | 14.1 (2.3) | 13.9 (2.1) | 14.1 (2.3) | 14.0 (2.2) |
| **Number of Office BP measurements, median (range)** | 2 (1―2) | 2 (1―2) | 2 (1―2) | 2 (1―2) |
| **Number of home BP measurements, median (range)** | 2 (1―7) | 3 (1―7) | 3 (1―7) | 2 (1―7) |
| **Office SBP before 20 weeks of gestation, mmHg** | 101 (8) | 106 (9) | 109 (9) | 116 (9) |
| **Office DBP before 20 weeks of gestation, mmHg** | 62 (7) | 64 (7) | 67 (7) | 71 (8) |
| **Office MAP before 20 weeks of gestation, mmHg** | 75 (7) | 78 (7) | 81 (7) | 86 (8) |
| **Home SBP before 20 weeks of gestation, mmHg** | 94 (4) | 101 (2) | 107 (2) | 116 (5) |
| **Home DBP before 20 weeks of gestation, mmHg** | 57 (5) | 61 (4) | 64 (5) | 69 (6) |
| **Home MAP before 20 weeks of gestation, mmHg** | 69 (4) | 74 (3) | 78 (3) | 85 (5) |
| **PMPCs, N (%)** | 25 (9.9) | 37 (15.5) | 41 (17.0) | 67 (27.5) |
| New-onset HDP, N (%) | 8 (3.2) | 19 (8.0) | 16 (6.6) | 49 (20.1) |
| Preeclampsia, N (%) | 3 (1.2) | 1 (0.4) | 5 (2.1) | 8 (3.3) |
| Gestational hypertension, N (%) | 5 (2.0) | 18 (7.6) | 11 (4.6) | 41 (16.8) |
| Placental abruption, N (%) | 1 (0.4) | 1 (0.4) | 1 (0.4) | 0 (0.0) |
| Preterm delivery (<37 weeks of gestation), N (%) | 3 (1.2) | 8 (3.4) | 9 (3.7) | 9 (3.7) |
| SGA infants (Birth weight <10th percentile), N (%) | 14 (5.6) | 15 (6.3) | 20 (8.3) | 16 (6.6) |
| Stillbirth at ≥28 weeks of gestation, N (%) | 1 (0.4) | 0 (0.0) | 1 (0.4) | 0 (0.0) |
| **Redefined PMPCs by replacing new-onset HDP with preeclampsia, N (%)** | 20 (7.9) | 22 (9.2) | 32 (13.3) | 29 (11.9) |
| **Infant sex, N (%)** |  |  |  |  |
| Male | 129 (51.2) | 126 (52.9) | 138 (57.3) | 114 (46.7) |
| Female | 123 (48.8) | 112 (47.1) | 103 (42.7) | 130 (53.3) |
| **Delivery week, weeks** | 39.7 (1.3) | 39.6 (1.7) | 39.5 (1.8) | 39.6 (1.9) |
| **Infant birth weight, g** | 3,090 (399) | 3,030 (417) | 3,040 (438) | 3,052 (429) |
| Low birth weight (<2,500 g), N (%) | 12 (4.8) | 21 (8.8) | 21 (8.7) | 16 (6.6) |

PMPCs include at least one of the following perinatal outcomes: new-onset HDP, placental abruption, preterm delivery, SGA infants, and stillbirth at ≥28 weeks of gestation.

Redefined PMPCs include at least one of the following perinatal outcomes: preeclampsia, placental abruption, preterm delivery, SGA infants, and stillbirth at ≥28 weeks of gestation.

Abbreviations: ART, assisted reproductive technology; BMI, body mass index; BP, blood pressure; DBP, diastolic blood pressure; HDP, hypertensive disorders of pregnancy; MAP, mean arterial pressure; PMPCs, placenta-mediated pregnancy complications; SBP, systolic blood pressure; s.d., standard deviation; SGA, small for gestational age.

**Supplementary Table 6. Characteristics according to quartiles of home DBP**

| **Variables** | **Participants according to quartiles of home DBP** | | | |
| --- | --- | --- | --- | --- |
|  | **Quartile 1 (46.0－57.8 mmHg), N=242** | **Quartile 2 (58.0－62.0 mmHg), N=242** | **Quartile 3 (62.2－67.0 mmHg), N=247** | **Quartile 4 (67.3－83.4 mmHg), N=244** |
| **Maternal age, years** | 31.2 (4.5) | 30.9 (5.0) | 31.2 (4.7) | 31.8 (4.7) |
| <35 years, N (%) | 181 (74.8) | 177 (73.1) | 186 (75.3) | 172 (70.5) |
| ≥35 years, N (%) | 61 (25.2) | 65 (26.9) | 59 (23.9) | 72 (29.5) |
| Missing, N (%) | 0 (0.0) | 0 (0.0) | 2 (0.8) | 0 (0.0) |
| **Height, cm** | 158.1 (5.2) | 158.6 (5.0) | 158.8 (5.1) | 158.4 (5.5) |
| **Pre-pregnancy body weight, kg** | 51.6 (6.6) | 53.3 (8.1) | 54.6 (7.9) | 56.5 (10.3) |
| **Pre-pregnancy BMI, kg/m²** | 20.6 (2.3) | 21.2 (2.9) | 21.7 (2.9) | 22.5 (3.9) |
| Underweight (<18.5 kg/m²), N (%) | 40 (16.5) | 32 (13.2) | 25 (10.1) | 27 (11.1) |
| Normal weight (18.5－24.9 kg/m²), N (%) | 194 (80.2) | 187 (77.3) | 191 (77.3) | 163 (66.8) |
| Overweight/Obese (≥25.0 kg/m²), N (%) | 8 (3.3) | 23 (9.5) | 31 (12.6) | 54 (22.1) |
| **Parity, N (%)** |  |  |  |  |
| Primipara | 144 (59.5) | 149 (61.6) | 154 (62.3) | 140 (57.4) |
| Multipara without HDP in a previous pregnancy | 93 (38.4) | 84 (34.7) | 86 (34.8) | 93 (38.1) |
| Multipara with HDP in a previous pregnancy | 2 (0.8) | 7 (2.9) | 3 (1.2) | 8 (3.3) |
| Missing | 3 (1.2) | 2 (0.8) | 4 (1.6) | 3 (1.2) |
| **ART, N (%)** | 3 (1.2) | 6 (2.5) | 9 (3.6) | 9 (3.7) |
| **Family history of hypertension, N (%)** | 11 (4.5) | 11 (4.5) | 13 (5.3) | 15 (6.1) |
| **Smoking status, N (%)** |  |  |  |  |
| No smoking before conception | 199 (82.2) | 216 (89.3) | 206 (83.4) | 203 (83.2) |
| Until conception was recognized | 33 (13.6) | 23 (9.5) | 33 (13.4) | 31 (12.7) |
| Smoking during pregnancy | 10 (4.1) | 3 (1.2) | 8 (3.2) | 10 (4.1) |
| **Alcohol drinking, N (%)** |  |  |  |  |
| No alcohol intake before conception | 107 (44.2) | 131 (54.1) | 139 (56.3) | 141 (57.8) |
| Until conception was recognized | 96 (39.7) | 95 (39.3) | 81 (32.8) | 77 (31.6) |
| Alcohol intake during pregnancy | 34 (14.0) | 14 (5.8) | 27 (10.9) | 24 (9.8) |
| Missing | 5 (2.1) | 2 (0.8) | 0 (0.0) | 2 (0.8) |
| **HbA1c before 20 weeks of gestation, %** | 5.0 (0.2) | 5.0 (0.2) | 5.0 (0.3) | 5.1 (0.2) |
| **Season of estimated date of confinement, N (%)** |  |  |  |  |
| Spring | 53 (21.9) | 62 (25.6) | 60 (24.3) | 67 (27.5) |
| Summer | 42 (17.4) | 58 (24.0) | 69 (27.9) | 77 (31.6) |
| Autumn | 59 (24.4) | 63 (26.0) | 64 (25.9) | 66 (27.0) |
| Winter | 88 (36.4) | 59 (24.4) | 54 (21.9) | 34 (13.9) |
| **Hyperthyroidism, N (%)** | 2 (0.8) | 0 (0.0) | 3 (1.2) | 2 (0.8) |
| **Hypothyroidism, N (%)** | 0 (0.0) | 2 (0.8) | 0 (0.0) | 0 (0.0) |
| **Chronic kidney disease, N (%)** | 0 (0.0) | 0 (0.0) | 0 (0.0) | 0 (0.0) |
| **Gestational age when Office BP was measured, weeks** | 14.1 (2.4) | 13.8 (2.3) | 13.9 (2.3) | 13.7 (2.2) |
| **Gestational age when home BP was measured, weeks** | 14.3 (2.3) | 13.9 (2.2) | 14.1 (2.2) | 13.9 (2.2) |
| **Number of Office BP measurements, median (range)** | 2 (1―2) | 2 (1―2) | 2 (1―2) | 2 (1―2) |
| **Number of home BP measurements, median (range)** | 3 (1―7) | 2 (1―7) | 3 (1―7) | 2 (1―7) |
| **Office SBP before 20 weeks of gestation, mmHg** | 103 (9) | 106 (9) | 108 (9) | 115 (10) |
| **Office DBP before 20 weeks of gestation, mmHg** | 60 (7) | 64 (6) | 67 (7) | 73 (7) |
| **Office MAP before 20 weeks of gestation, mmHg** | 75 (7) | 78 (6) | 80 (7) | 87 (7) |
| **Home SBP before 20 weeks of gestation, mmHg** | 97 (7) | 102 (6) | 105 (6) | 113 (7) |
| **Home DBP before 20 weeks of gestation, mmHg** | 54 (3) | 60 (1) | 64 (1) | 72 (4) |
| **Home MAP before 20 weeks of gestation, mmHg** | 68 (3) | 74 (2) | 78 (2) | 86 (4) |
| **PMPCs, N (%)** | 16 (6.6) | 37 (15.3) | 47 (19.0) | 70 (28.7) |
| New-onset HDP, N (%) | 8 (3.3) | 14 (5.8) | 23 (9.3) | 47 (19.3) |
| Preeclampsia, N (%) | 1 (0.4) | 4 (1.7) | 6 (2.4) | 6 (2.5) |
| Gestational hypertension, N (%) | 7 (2.9) | 10 (4.1) | 17 (6.9) | 41 (16.8) |
| Placental abruption, N (%) | 0 (0.0) | 3 (1.2) | 0 (0.0) | 0 (0.0) |
| Preterm delivery (<37 weeks of gestation), N (%) | 0 (0.0) | 9 (3.7) | 7 (2.8) | 13 (5.3) |
| SGA infants (Birth weight <10th percentile), N (%) | 9 (3.7) | 15 (6.2) | 21 (8.5) | 20 (8.2) |
| Stillbirth at ≥28 weeks of gestation, N (%) | 0 (0.0) | 0 (0.0) | 2 (0.8) | 0 (0.0) |
| **Redefined PMPCs by replacing new-onset HDP with preeclampsia, N (%)** | 10 (4.1) | 28 (11.6) | 31 (12.6) | 34 (13.9) |
| **Infant sex, N (%)** |  |  |  |  |
| Male | 114 (47.1) | 133 (55.0) | 121 (49.0) | 139 (57.0) |
| Female | 128 (52.9) | 109 (45.0) | 126 (51.0) | 105 (43.0) |
| **Delivery week, weeks** | 39.6 (1.1) | 39.6 (1.7) | 39.7 (1.6) | 39.4 (2.2) |
| **Infant birth weight, g** | 3,095 (367) | 3,052 (415) | 3,039 (430) | 3,029 (463) |
| Low birth weight (<2,500 g), N (%) | 8 (3.3) | 17 (7.0) | 24 (9.7) | 21 (8.6) |

PMPCs include at least one of the following perinatal outcomes: new-onset HDP, placental abruption, preterm delivery, SGA infants, and stillbirth at ≥28 weeks of gestation.

Redefined PMPCs include at least one of the following perinatal outcomes: preeclampsia, placental abruption, preterm delivery, SGA infants, and stillbirth at ≥28 weeks of gestation.

Abbreviations: ART, assisted reproductive technology; BMI, body mass index; BP, blood pressure; DBP, diastolic blood pressure; HDP, hypertensive disorders of pregnancy; MAP, mean arterial pressure; PMPCs, placenta-mediated pregnancy complications; SBP, systolic blood pressure; s.d., standard deviation; SGA, small for gestational age.

**Supplementary Table 7. Characteristics according to quartiles of home MAP**

| **Variables** | **Participants according to quartiles of home MAP** | | | |
| --- | --- | --- | --- | --- |
|  | **Quartile 1 (58.7－71.5 mmHg), N=242** | **Quartile 2 (71.5－76.0 mmHg), N=245** | **Quartile 3 (76.1－81.0 mmHg), N=246** | **Quartile 4 (81.1－97.8 mmHg), N=242** |
| **Maternal age, years** | 31.2 (4.3) | 30.8 (4.9) | 31.3 (4.8) | 31.8 (4.9) |
| <35 years, N (%) | 185 (76.4) | 185 (75.5) | 177 (72.0) | 169 (69.8) |
| ≥35 years, N (%) | 57 (23.6) | 59 (24.1) | 68 (27.6) | 73 (30.2) |
| Missing, N (%) | 0 (0.0) | 1 (0.4) | 1 (0.4) | 0 (0.0) |
| **Height, cm** | 158.5 (5.1) | 158.2 (4.9) | 158.8 (5.2) | 158.5 (5.5) |
| **Pre-pregnancy body weight, kg** | 51.2 (6.2) | 53.3 (7.7) | 55.0 (8.5) | 56.6 (10.3) |
| **Pre-pregnancy BMI, kg/m²** | 20.4 (2.1) | 21.3 (2.9) | 21.8 (3.0) | 22.5 (3.9) |
| Underweight (<18.5 kg/m²), N (%) | 44 (18.2) | 32 (13.1) | 20 (8.1) | 28 (11.6) |
| Normal weight (18.5－24.9 kg/m²), N (%) | 193 (79.8) | 188 (76.7) | 195 (79.3) | 159 (65.7) |
| Overweight/Obese (≥25.0 kg/m²), N (%) | 5 (2.1) | 25 (10.2) | 31 (12.6) | 55 (22.7) |
| **Parity, N (%)** |  |  |  |  |
| Primipara | 149 (61.6) | 150 (61.2) | 151 (61.4) | 137 (56.6) |
| Multipara without HDP in a previous pregnancy | 90 (37.2) | 85 (34.7) | 87 (35.4) | 94 (38.8) |
| Multipara with HDP in a previous pregnancy | 1 (0.4) | 7 (2.9) | 4 (1.6) | 8 (3.3) |
| Missing | 2 (0.8) | 3 (1.2) | 4 (1.6) | 3 (1.2) |
| **ART, N (%)** | 4 (1.7) | 3 (1.2) | 10 (4.1) | 10 (4.1) |
| **Family history of hypertension, N (%)** | 10 (4.1) | 13 (5.3) | 14 (5.7) | 13 (5.4) |
| **Smoking status, N (%)** |  |  |  |  |
| No smoking before conception | 208 (86.0) | 210 (85.7) | 207 (84.1) | 199 (82.2) |
| Until conception was recognized | 27 (11.2) | 30 (12.2) | 32 (13.0) | 31 (12.8) |
| Smoking during pregnancy | 7 (2.9) | 5 (2.0) | 7 (2.8) | 12 (5.0) |
| **Alcohol drinking, N (%)** |  |  |  |  |
| No alcohol intake before conception | 116 (47.9) | 119 (48.6) | 144 (58.5) | 139 (57.4) |
| Until conception was recognized | 95 (39.3) | 95 (38.8) | 84 (34.1) | 75 (31.0) |
| Alcohol intake during pregnancy | 26 (10.7) | 29 (11.8) | 18 (7.3) | 26 (10.7) |
| Missing | 5 (2.1) | 2 (0.8) | 0 (0.0) | 2 (0.8) |
| **HbA1c before 20 weeks of gestation, %** | 5.0 (0.3) | 5.0 (0.2) | 5.0 (0.2) | 5.0 (0.3) |
| **Season of estimated date of confinement, N (%)** |  |  |  |  |
| Spring | 52 (21.5) | 61 (24.9) | 58 (23.6) | 71 (29.3) |
| Summer | 43 (17.8) | 57 (23.3) | 67 (27.2) | 79 (32.6) |
| Autumn | 57 (23.6) | 66 (26.9) | 66 (26.8) | 63 (26.0) |
| Winter | 90 (37.2) | 61 (24.9) | 55 (22.4) | 29 (12.0) |
| **Hyperthyroidism, N (%)** | 2 (0.8) | 1 (0.4) | 2 (0.8) | 2 (0.8) |
| **Hypothyroidism, N (%)** | 0 (0.0) | 2 (0.8) | 0 (0.0) | 0 (0.0) |
| **Chronic kidney disease, N (%)** | 0 (0.0) | 0 (0.0) | 0 (0.0) | 0 (0.0) |
| **Gestational age when office BP was measured, weeks** | 14.0 (2.3) | 13.9 (2.3) | 13.7 (2.3) | 13.8 (2.2) |
| **Gestational age when home BP was measured, weeks** | 14.2 (2.2) | 14.1 (2.3) | 13.9 (2.2) | 14.0 (2.2) |
| **Number of Office BP measurements, median (range)** | 2 (1―2) | 2 (1―2) | 2 (1―2) | 2 (1―2) |
| **Number of home BP measurements, median (range)** | 3 (1―7) | 3 (1―7) | 3 (1―7) | 2 (1―7) |
| **Office SBP before 20 weeks of gestation, mmHg** | 102 (9) | 106 (8) | 109 (9) | 115 (10) |
| **Office DBP before 20 weeks of gestation, mmHg** | 61 (7) | 64 (7) | 67 (7) | 72 (8) |
| **Office MAP before 20 weeks of gestation, mmHg** | 74 (7) | 78 (6) | 81 (7) | 87 (7) |
| **Home SBP before 20 weeks of gestation, mmHg** | 95 (5) | 101 (4) | 106 (4) | 114 (6) |
| **Home DBP before 20 weeks of gestation, mmHg** | 55 (3) | 60 (2) | 64 (3) | 72 (4) |
| **Home MAP before 20 weeks of gestation, mmHg** | 68 (3) | 74 (1) | 78 (1) | 86 (4) |
| **PMPCs, N (%)** | 13 (5.4) | 40 (16.3) | 42 (17.1) | 75 (31.0) |
| New-onset HDP, N (%) | 4 (1.7) | 16 (6.5) | 22 (8.9) | 50 (20.7) |
| Preeclampsia, N (%) | 2 (0.8) | 3 (1.2) | 4 (1.6) | 8 (3.3) |
| Gestational hypertension, N (%) | 2 (0.8) | 13 (5.3) | 18 (7.3) | 42 (17.4) |
| Placental abruption, N (%) | 0 (0.0) | 3 (1.2) | 0 (0.0) | 0 (0.0) |
| Preterm delivery (<37 weeks of gestation), N (%) | 1 (0.4) | 10 (4.1) | 6 (2.4) | 12 (5.0) |
| SGA infants (Birth weight <10th percentile), N (%) | 8 (3.3) | 17 (6.9) | 16 (6.5) | 24 (9.9) |
| Stillbirth at ≥28 weeks of gestation, N (%) | 0 (0.0) | 1 (0.4) | 1 (0.4) | 0 (0.0) |
| **Redefined PMPCs by replacing new-onset HDP with preeclampsia, N (%)** | 11 (4.5) | 29 (11.8) | 25 (10.2) | 38 (15.7) |
| **Infant sex, N (%)** |  |  |  |  |
| Male | 122 (50.4) | 130 (53.1) | 122 (49.6) | 133 (55.0) |
| Female | 120 (49.6) | 115 (46.9) | 124 (50.4) | 109 (45.0) |
| **Delivery week, weeks** | 39.6 (1.1) | 39.5 (2.0) | 39.7 (1.4) | 39.5 (2.0) |
| **Infant birth weight, g** | 3,089 (356) | 3,049 (465) | 3,048 (400) | 3,028 (452) |
| Low birth weight (<2,500 g), N (%) | 9 (3.7) | 19 (7.8) | 21 (8.5) | 21 (8.7) |

PMPCs include at least one of the following perinatal outcomes: new-onset HDP, placental abruption, preterm delivery, SGA infants, and stillbirth at ≥28 weeks of gestation.

Redefined PMPCs include at least one of the following perinatal outcomes: preeclampsia, placental abruption, preterm delivery, SGA infants, and stillbirth at ≥28 weeks of gestation.

Abbreviations: ART, assisted reproductive technology; BMI, body mass index; BP, blood pressure; DBP, diastolic blood pressure; HDP, hypertensive disorders of pregnancy; MAP, mean arterial pressure; PMPCs, placenta-mediated pregnancy complications; SBP, systolic blood pressure; s.d., standard deviation; SGA, small for gestational age.

**Supplementary Table 8. Comparison of the associations between SBP and DBP with PMPCs (Likelihood ratio test)**

| **OBP or HBP before 20 weeks of gestation** | **Model 1 Unadjusted model** | | **Model 2 Adjusted model** | |
| --- | --- | --- | --- | --- |
|  | **Likelihood ratio test statistic** | **P-value** | **D3-statistic** | **P-value** |
| **Office BP, per 1s.d. increase** |  |  |  |  |
| Office SBP + office DBP vs. Office SBP | 27.84 | <0.0001 | 25.17 | <0.0001 |
| Office SBP + office DBP vs. Office DBP | 0.19 | 0.7 | 0.04 | 0.8 |
| **Home BP, per 1s.d. increase** |  |  |  |  |
| Home SBP + home DBP vs. Home SBP | 21.53 | <0.0001 | 19.97 | <0.0001 |
| Home SBP + home DBP vs. Home DBP | 0.85 | 0.4 | 1.92 | 0.2 |

The D3-statistic is a pooled likelihood ratio test statistic across multiple imputations.

Model 1: Unadjusted model

Model 2: Adjusted for maternal age, pre-pregnancy BMI, parity, ART, family history of hypertension, smoking status, alcohol intake, HbA1c level before 20 weeks of gestation, season of EDC, infant sex, and gestational age when OBP was measured.

The comparison between MAP and SBP or DBP could not be conducted because of multicollinearity.

Abbreviations: ART, assisted reproductive technology; BP, blood pressure; BMI, body mass index; DBP, diastolic BP; EDC, estimated date of confinement; HBP, home BP; MAP, mean arterial pressure; OBP, office BP; PMPC, placenta-mediated pregnancy complications; SBP, systolic BP.

1s.d. = 10 mmHg for office SBP, 8 mmHg for office DBP, and 8 mmHg for office MAP.

1s.d. = 9 mmHg for home SBP, 7 mmHg for home DBP, and 7 mmHg for home MAP.

**Supplementary Table 9. Comparison of the associations between OBP and HBP with the redefined PMPCs, in which new-onset HDP was replaced by preeclampsia**

| **OBP and/or HBP before 20 weeks of gestation** | **Model 1 Unadjusted model** | | **Model 2 Adjusted model** | |
| --- | --- | --- | --- | --- |
|  | **Likelihood ratio test statistic** | **P-value** | **D3-statistic** | **P-value** |
| **SBP, per 1s.d. increase** |  |  |  |  |
| Office SBP + home SBP vs. Office SBP | 9.08 | 0.003 | 8.10 | 0.004 |
| Office SBP + home SBP vs. Home SBP | 1.68 | 0.2 | 1.19 | 0.3 |
| **DBP, per 1s.d. increase** |  |  |  |  |
| Office DBP + home DBP vs. Office DBP | 6.93 | 0.01 | 6.61 | 0.01 |
| Office DBP + home DBP vs. Home DBP | 0.39 | 0.5 | 0.44 | 0.5 |
| **MAP, per 1s.d. increase** |  |  |  |  |
| Office MAP + home MAP vs. Office MAP | 9.07 | 0.003 | 8.66 | 0.003 |
| Office MAP + home MAP vs. Home MAP | 0.06 | 0.8 | 0.04 | 0.9 |

The D3-statistic is a pooled likelihood ratio test statistic across multiple imputations.

Redefined PMPCs include at least one of the following perinatal outcomes: preeclampsia, placental abruption, preterm delivery, SGA infants, and stillbirth at ≥28 weeks of gestation.

Model 1: Unadjusted model

Model 2: Adjusted for maternal age, pre-pregnancy BMI, parity, ART, family history of hypertension, smoking status, alcohol intake, HbA1c level before 20 weeks of gestation, season of EDC, infant sex, and gestational age when OBP was measured.

Abbreviations: ART, assisted reproductive technology; BP, blood pressure; BMI, body mass index; DBP, diastolic BP; EDC, estimated date of confinement; HBP, home BP; MAP, mean arterial pressure; OBP, office BP; PMPCs, placenta–mediated pregnancy complications; SBP, systolic BP.

1s.d. = 10 mmHg for office SBP and 8 mmHg for office DBP.

1s.d. = 9 mmHg for home SBP and 7 mmHg for home DBP.

**Supplementary Table 10. Comparison of the associations between SBP and DBP with the redefined PMPCs, in which new–onset HDP replaced by preeclampsia (Likelihood ratio test)**

| **OBP or HBP before 20 weeks of gestation** | **Model 1 Unadjusted model** | | **Model 2 Adjusted model** | |
| --- | --- | --- | --- | --- |
|  | **Likelihood ratio test statistic** | **P-value** | **D3-statistic** | **P-value** |
| **Office BP, per 1s.d. increase** |  |  |  |  |
| Office SBP + office DBP vs. Office SBP | 11.36 | 0.001 | 9.96 | 0.002 |
| Office SBP + office DBP vs. Office DBP | 4.25 | 0.04 | 3.55 | 0.06 |
| **Home BP, per 1s.d. increase** |  |  |  |  |
| Home SBP + home DBP vs. Home SBP | 6.25 | 0.01 | 5.66 | 0.02 |
| Home SBP + home DBP vs. Home DBP | 0.006 | 0.9 | 0.04 | 0.9 |

The D3-statistic is a pooled likelihood ratio test statistic across multiple imputations.

Redefined PMPCs include at least one of the following perinatal outcomes: preeclampsia, placental abruption, preterm delivery, SGA infants, and stillbirth at ≥28 weeks of gestation.

Model 1: Unadjusted model

Model 2: Adjusted for maternal age, pre-pregnancy BMI, parity, ART, family history of hypertension, smoking status, alcohol intake, HbA1c level before 20 weeks of gestation, season of EDC, infant sex, and gestational age when OBP was measured.

The comparison between MAP and SBP or DBP could not be conducted because of multicollinearity.

Abbreviations: ART, assisted reproductive technology; BP, blood pressure; BMI, body mass index; DBP, diastolic BP; EDC, estimated date of confinement; HBP, home BP; MAP, mean arterial pressure; OBP, office BP; PMPCs, placenta-mediated pregnancy complications; SBP, systolic BP.

1s.d. = 10 mmHg for office SBP and 8 mmHg for office DBP.

1s.d. = 9 mmHg for home SBP and 7 mmHg for home DBP.

**Supplementary Figure 1. Directed acyclic graph of the associations of OBP and HBP with PMPCs.**


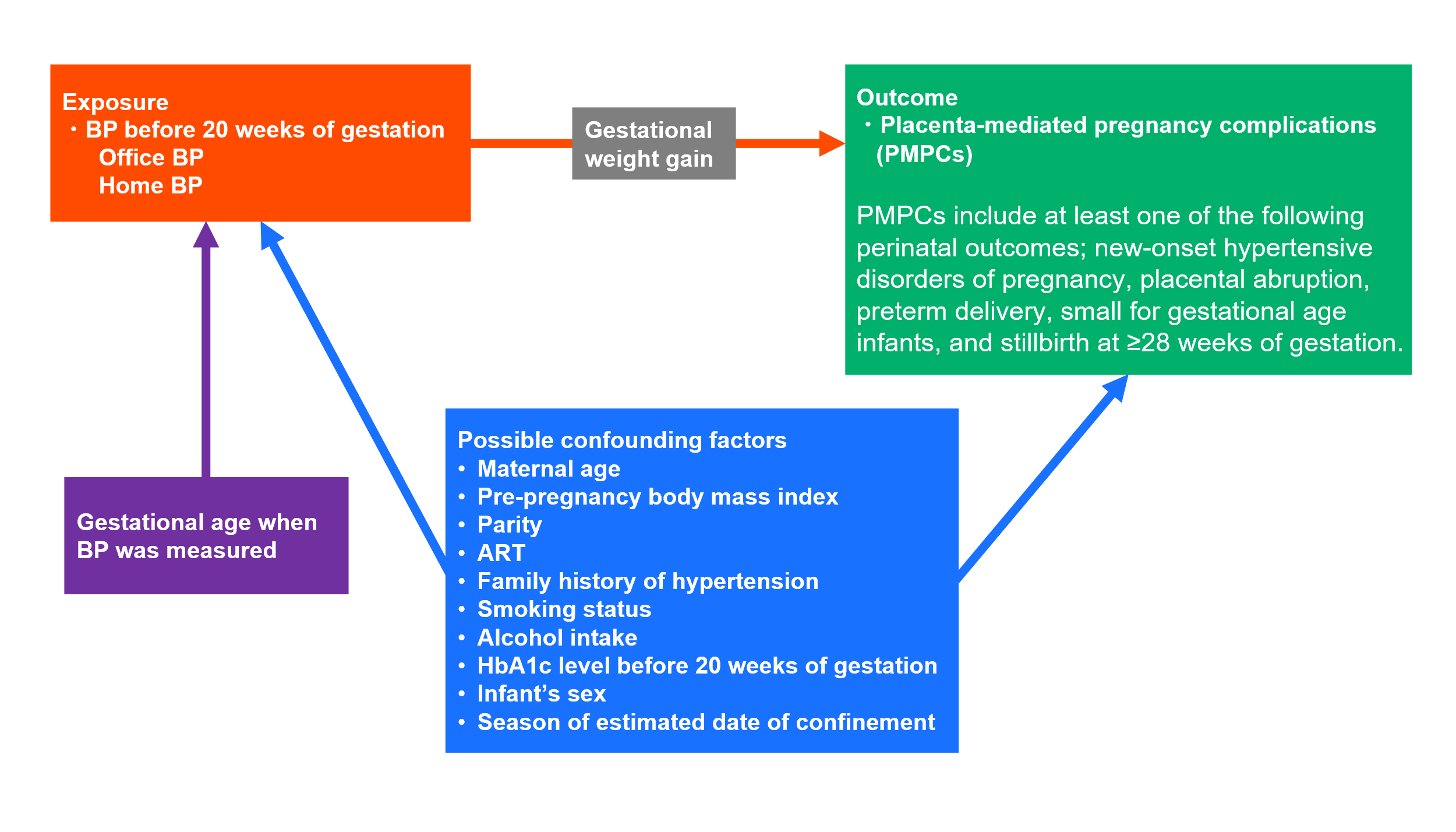


PMPCs include at least one of the following perinatal outcomes: new-onset HDP, placental abruption, preterm delivery, SGA infants, and stillbirth at ≥28 weeks of gestation.

Abbreviations: ART, assisted reproductive technology; BP, blood pressure; OBP, office BP; HBP, home BP; HDP, hypertensive disorders of pregnancy; PMPCs, placenta-mediated pregnancy complications; SGA, small for gestational age.

**Supplementary Figure 2. Comparison of the associations between SBP and DBP with PMPCs (Simultaneous analysis)**


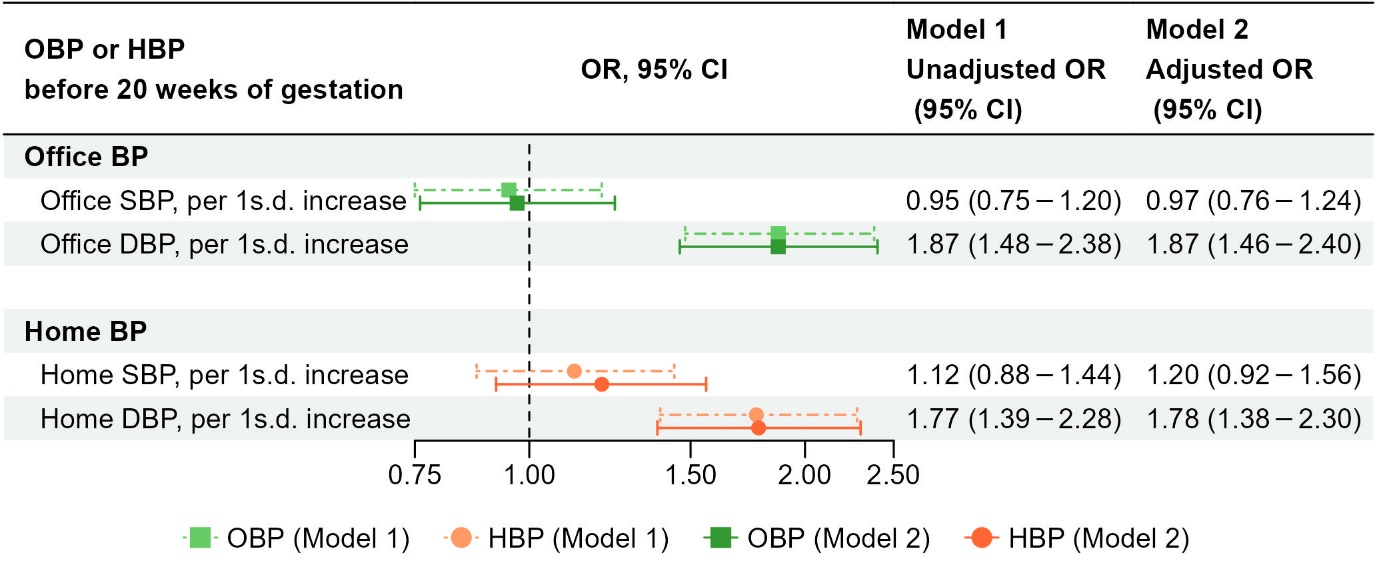


PMPCs include at least one of the following perinatal outcomes: new-onset HDP, placental abruption, preterm delivery, SGA infants, and stillbirth at ≥28 weeks of gestation.

Simultaneous analysis indicates that each SBP and DBP was included in a binary logistic regression model, as continuous variables simultaneously per 1s.d..

Model 1: Unadjusted model

Model 2: Adjusted for maternal age, pre-pregnancy BMI, parity, ART, family history of hypertension, smoking status, alcohol intake, HbA1c level before 20 weeks of gestation, season of EDC, infant sex, and gestational age when OBP was measured.

The comparison between MAP and SBP or DBP could not be conducted because of multicollinearity.

Abbreviations: ART, assisted reproductive technology; BP, blood pressure; BMI, body mass index; CI, confidence interval; DBP, diastolic BP; EDC, estimated date of confinement; HBP, home BP; MAP, mean arterial pressure; OBP, office BP; PMPCs, placenta-mediated pregnancy complications; SBP, systolic BP.

1s.d. = 10 mmHg for office SBP and 8 mmHg for office DBP.

1s.d. = 9 mmHg for home SBP and 7 mmHg for home DBP.

**Supplementary Figure 3. The association between OBP and the redefined PMPCs, in which new-onset HDP was replaced by preeclampsia**

**
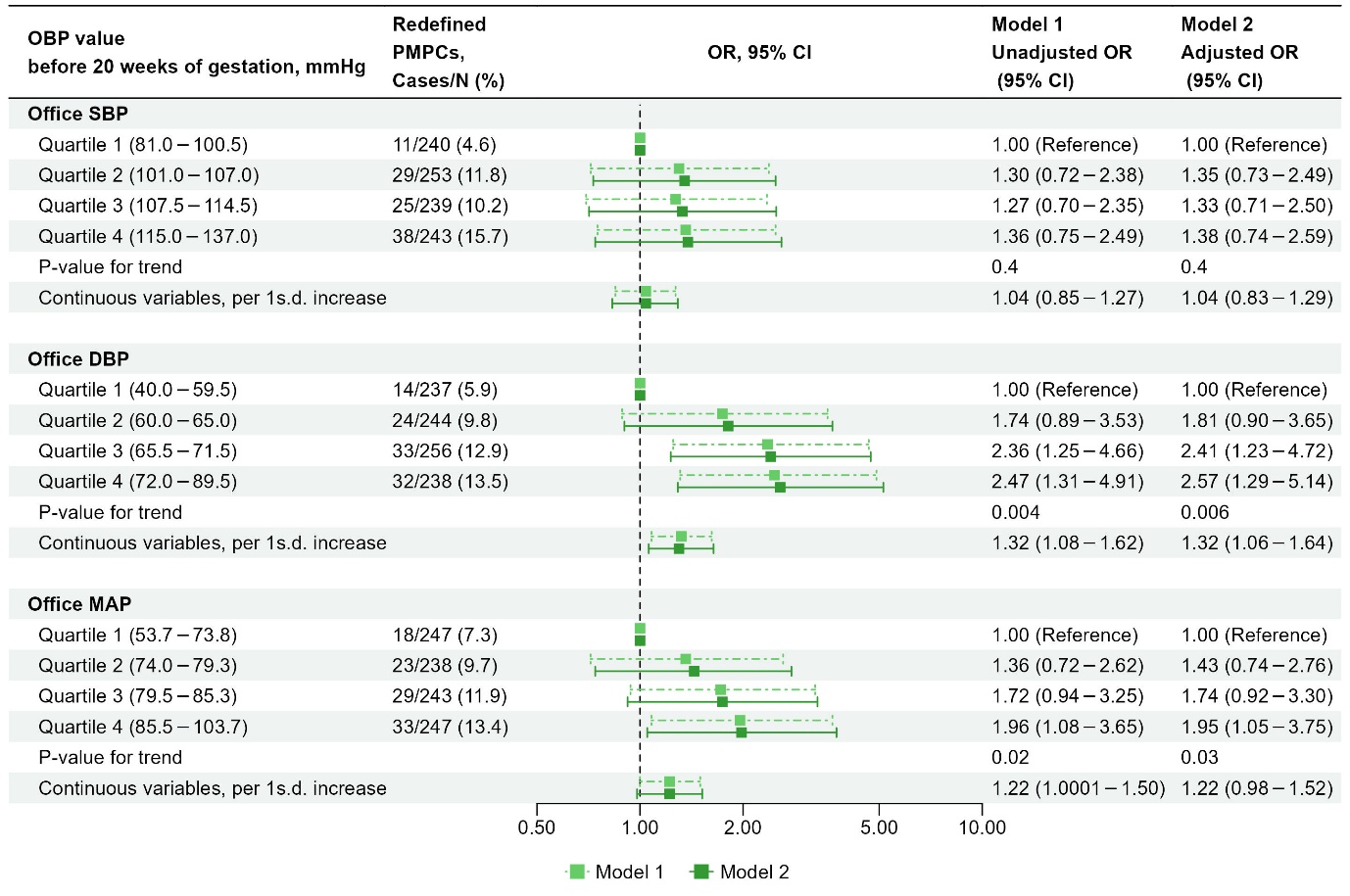
**

Redefined PMPCs include at least one of the following perinatal outcomes: preeclampsia, placental abruption, preterm delivery, SGA infants, and stillbirth at ≥28 weeks of gestation.

Model 1: Unadjusted model

Model 2: Adjusted for maternal age, pre-pregnancy BMI, parity, ART, family history of hypertension, smoking status, alcohol intake, HbA1c level before 20 weeks of gestation, season of EDC, infant sex, and gestational age when OBP was measured.

Abbreviations: ART, assisted reproductive technology; BP, blood pressure; BMI, body mass index; CI, confidence interval; DBP, diastolic BP; EDC, estimated date of confinement; HDP, hypertensive disorders of pregnancy; MAP, mean arterial pressure; OBP, office BP; OR, odds ratio; PMPCs, placenta-mediated pregnancy complications; SBP, SBP, systolic BP; s.d., standard deviation; SGA, small for gestational age.

1s.d. = 10 mmHg for office SBP, 8 mmHg for office DBP and 8 mmHg for office MAP.

**Supplementary Figure 4. The association between HBP and the redefined PMPCs, in which new-onset HDP was replaced by preeclampsia**


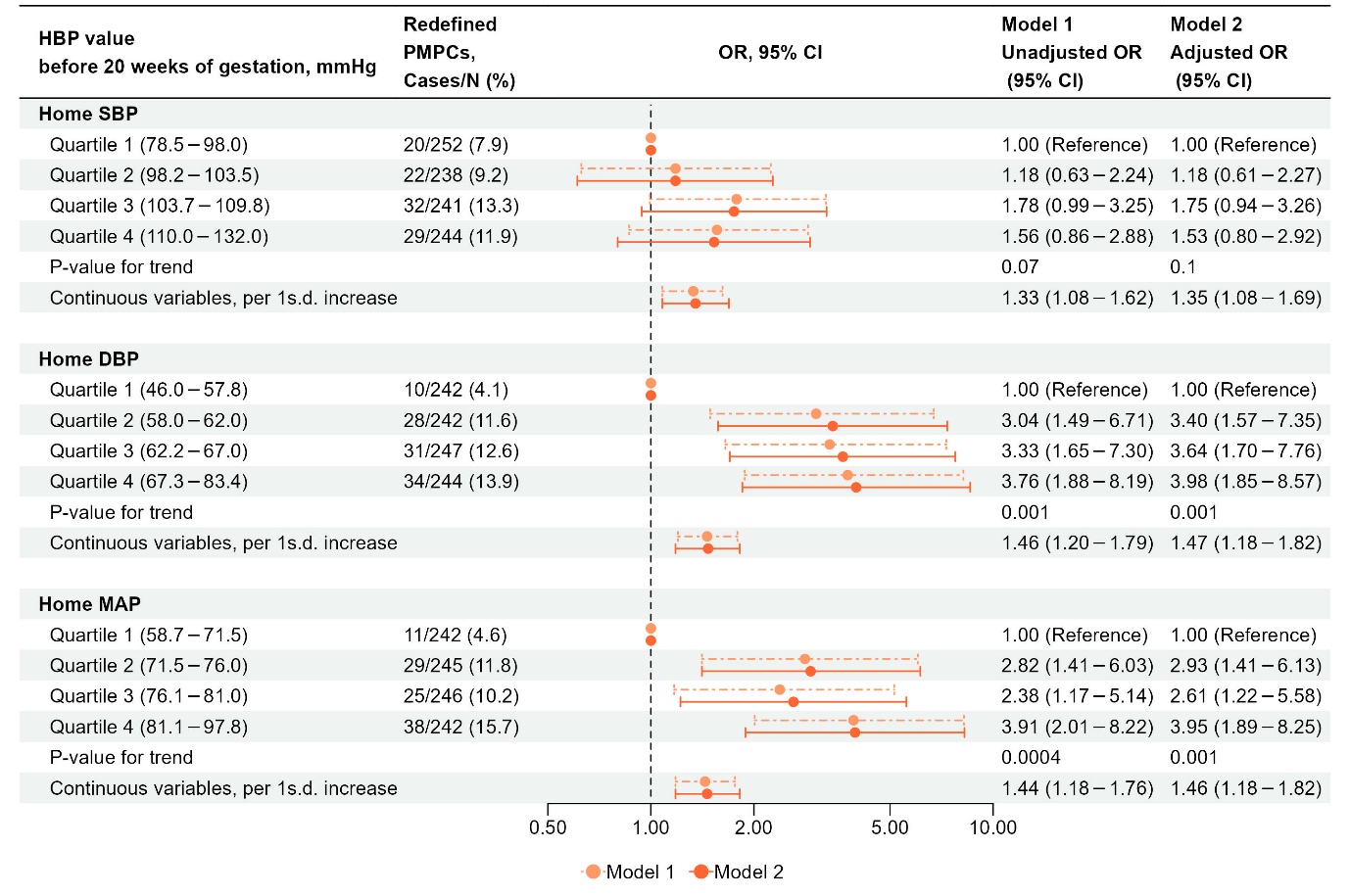


Redefined PMPCs include at least one of the following perinatal outcomes: preeclampsia, placental abruption, preterm delivery, SGA infants, and stillbirth at ≥28 weeks of gestation.

Model 1: Unadjusted model

Model 2: Adjusted for maternal age, pre-pregnancy BMI, parity, ART, family history of hypertension, smoking status, alcohol intake, HbA1c level before 20 weeks of gestation, season of EDC, infant sex, and gestational age when HBP was measured.

Abbreviations: ART, assisted reproductive technology; BP, blood pressure; BMI, body mass index; CI, confidence interval; DBP, diastolic BP; EDC, estimated date of confinement; HBP, home BP; HDP, hypertensive disorders of pregnancy; MAP, mean arterial pressure; OR, odds ratio; PMPCs, placenta-mediated pregnancy complications; SBP, systolic BP; s.d., standard deviation; SGA, small for gestational age.

1s.d. = 9 mmHg for home SBP, 7 mmHg for home DBP and 7 mmHg for home MAP.

**Supplementary Figure 5. Comparison of the associations between OBP and HBP with the redefined PMPCs, in which new-onset HDP was replaced by preeclampsia (Simultaneous analysis)**


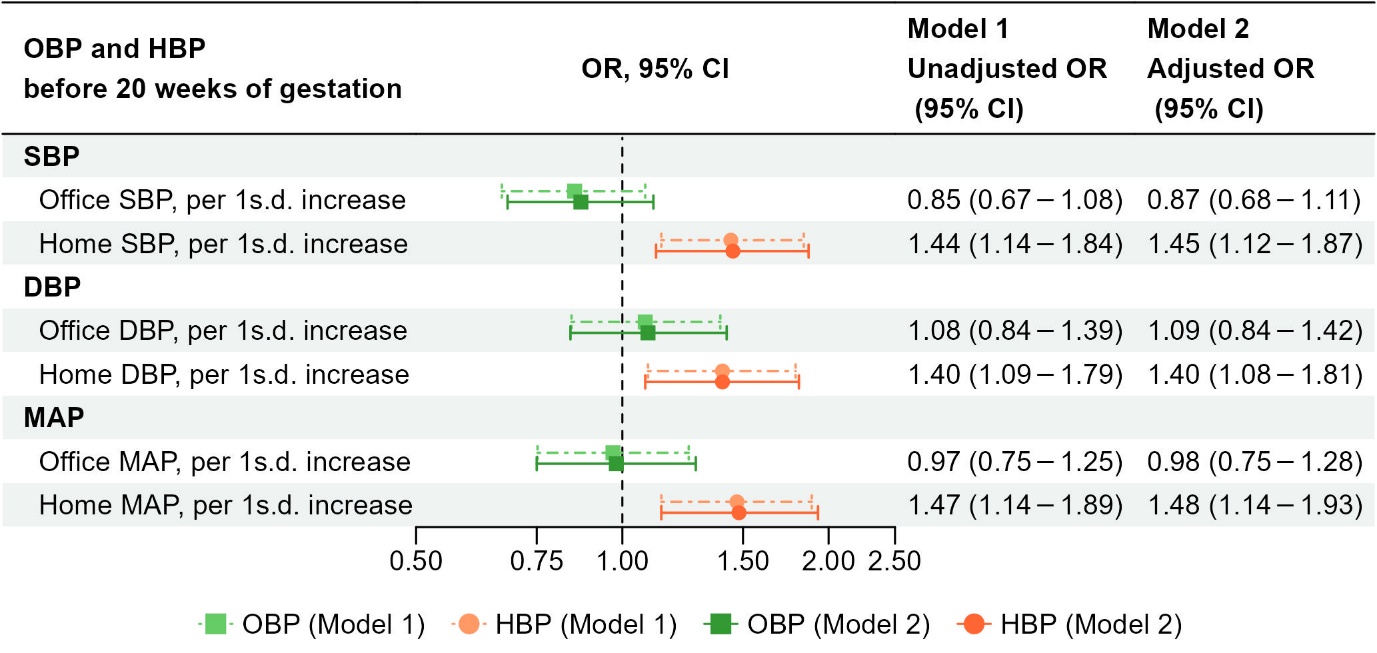


Redefined PMPCs include at least one of the following perinatal outcomes; preeclampsia, placental abruption, preterm delivery, SGA infants, and stillbirth at ≥28 weeks of gestation.

Simultaneous analysis indicates that each OBP and HBP were included into a binary logistic regression model, as continuous variables simultaneously per 1s.d..

Model 1: Unadjusted model

Model 2: Adjusted for maternal age, pre-pregnancy BMI, parity, ART, family history of hypertension, smoking status, alcohol intake, HbA1c level before 20 weeks of gestation, season of EDC, infant sex, and gestational age when OBP was measured.

Abbreviations: ART, assisted reproductive technology; BP, blood pressure; BMI, body mass index; CI, confidence interval; DBP, diastolic BP; EDC, estimated date of confinement; HBP, home BP; HDP, hypertensive disorders of pregnancy; MAP, mean arterial pressure; OBP, office BP; OR, odds ratio; PMPCs, placenta-mediated pregnancy complications; SBP, systolic BP; s.d., standard deviation.

1s.d. = 10 mmHg for office SBP, 8 mmHg for office DBP and 8 mmHg for office MAP.

1s.d. = 9 mmHg for home SBP, 7 mmHg for home DBP and 7 mmHg for home MAP.

**Supplementary Figure 6. Comparison of the associations between SBP and DBP with the redefined PMPCs, in which new-onset HDP was replaced by preeclampsia (Simultaneous analysis)**


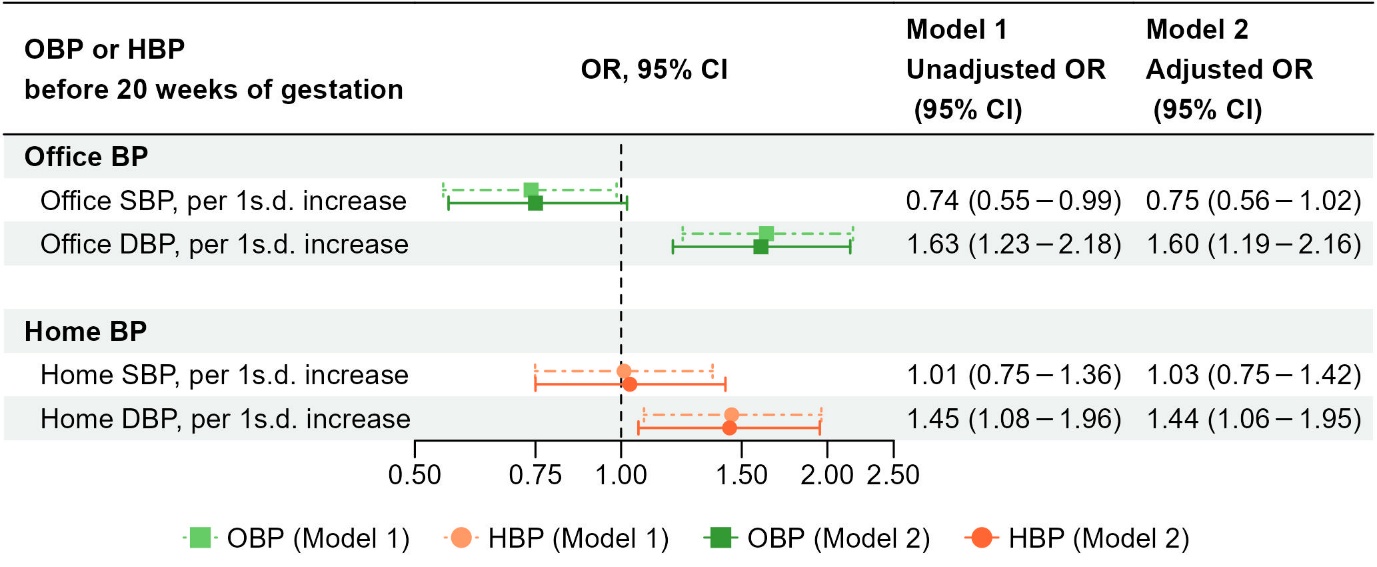


Redefined PMPCs include at least one of the following perinatal outcomes; preeclampsia, placental abruption, preterm delivery, SGA infants, and stillbirth at ≥28 weeks of gestation.

Simultaneous analysis indicates that each SBP and DBP were included into a binary logistic regression model, as continuous variables simultaneously per 1s.d..

Model 1: Unadjusted model

Model 2: Adjusted for maternal age, pre–pregnancy BMI, parity, ART, family history of hypertension, smoking status, alcohol intake, HbA1c level before 20 weeks of gestation, season of EDC, infant sex, and gestational age when OBP was measured.

The comparison between MAP and SBP or DBP could not be conducted because of multicollinearity.

Abbreviations: ART, assisted reproductive technology; BP, blood pressure; BMI, body–mass index; CI, confidence interval; DBP, diastolic BP; EDC, estimated date of confinement; HBP, home BP; MAP, mean arterial pressure; OBP, office BP; PMPCs, placenta–mediated pregnancy complications; SBP, systolic BP.

1s.d. = 10 mmHg for office SBP and 8 mmHg for office DBP.

1s.d. = 9 mmHg for home SBP and 7 mmHg for home DBP.
